# Different Metabolites in the Roots, Seeds, and Leaves of *Acanthopanax senticosus* and Their Role in Alleviating Oxidative Stress

**DOI:** 10.1155/2021/6628880

**Published:** 2021-04-15

**Authors:** Jie Su, Qi Wang, Zhifeng Li, Yan Feng, Yan Li, Shinlin Yang, Yulin Feng

**Affiliations:** ^1^Jiangxi University of Traditional Chinese Medicine, Nanchang 330002, China; ^2^State Key Laboratory of Innovative Drug and Efficient Energy-Saving Pharmaceutical Equipment, Nanchang 330006, China; ^3^Nanchang Key Laboratory of Active Ingredients of Traditional Chinese Medicine and Natural Medicine, Nanchang 330006, China

## Abstract

In this study, we examined the metabolites from different parts of *Acanthopanax senticosus* and their role in alleviating damage caused by oxidative stress. We used UHPLC-QTOF-MS to analyze the chemical components in the root, seed, and leaf extracts of *A. senticosus*. Two multivariate statistical analysis methods—namely, principal component analysis and partial least square discriminant analysis—were used to distinguish the samples obtained from different parts of the plant. Using univariate statistics, 130 different metabolites were screened out. Among these, the relative content of flavonoids and terpenoids was found to be highest in the leaves, the lignin and phenolic acid content was highest in the roots, and the amino acid and phenolic acid levels were highest in seeds. An MTT assay was used to test the anti-H_2_O_2_ oxidative damage to PC12 cells in different parts of the sample. Lastly, using Pearson's correlation analysis, various metabolites from different parts of *A. senticosus* were correlated with their antioxidant effects from the corresponding parts. Fifty-two related different metabolites were found, of which 20 metabolites that were positively correlated to oxidative stress were present at a relatively higher level in the roots, whereas 32 metabolites that were negatively correlated were present at relatively higher levels in the seeds and leaves. The results of this study reveal the distribution characteristics and the antioxidant activity of different metabolites of *A. senticosus* and provide a reference for the rational development of its medicinal parts.

## 1. Introduction

With an increase in the aging phenomenon, millions of individuals worldwide are affected by neurodegenerative diseases, which is one of the most significant challenges faced by modern society [1]. In particular, Alzheimer's disease (AD), Parkinson's disease (PD), and amyotrophic lateral sclerosis (ALS) are rampant. AD affects nearly 50 million people worldwide and is expected to grow steadily in the next decade. Although the annual medical expenses for neurodegenerative diseases are high, there is currently no definitive cure [2]. Therefore, research on the prevention and treatment of neurodegenerative diseases is warranted owing to the huge medical and public health burden that is associated with neurodegenerative diseases.

Oxidative stress injury as one of the breakthrough points of neurodegenerative diseases has received extensive attention in recent years. Oxidative stress is a phenomenon attributed to an imbalance in the production and removal of oxygen-reactive substances in cells [3]. Oxidative stress injury produces a large number of free radicals, which damages cells and tissues by destroying the integrity of the cell membrane by activating apoptosis-related signaling pathways and eventually inducing apoptosis [4]. Free radicals produced because of oxidative stress can damage neurons and result in neurodegenerative disorders. Neuronal cells have a stronger dependence and demand for energy resulting from oxidative phosphorylation, and they themselves are easier to be oxidized because they contain more polyunsaturated fatty acids than other tissues. Moreover, the concentration of antioxidant enzymes is relatively low and other factors render the neurons being highly sensitive to oxidative stress [5]. Several studies have shown that oxidative stress is closely related to the pathogenesis of neurodegenerative diseases. Activation of glial cells and an excess of reactive oxygen species can cause protein misfolding and mitochondrial dysfunction, in turn leading to apoptosis [6, 7]. PC12 cells are widely used in the establishment of neuronal damage-related models owing to their high structural and functional similarity with dopaminergic neurons. These cells are, therefore, used to study neurodegenerative diseases such as AD and PD [8]. The primary methods used to develop this model include H_2_O_2_ damage or glutamate damage [9]. In our study, a model of oxidative stress injury was induced by H_2_O_2_ in PC12 cells and used for subsequent experiments.

*Acanthopanax senticosus* is a traditional Chinese medicine rich in saponins, flavonoids, and phenolic acids [10]. It plays an important role in the treatment of diseases of the central nervous system [11]. For example, *A. senticosus* is indicated as auxiliary treatment for vascular dementia, cerebral ischemia-reperfusion injury, depression, and PD among other conditions [12, 13]. Studies report that the polysaccharide of *A. senticosus* has protective effects on H_2_O_2_-injured hippocampal nerve cells. However, the primary compound of *A. senticosus* that is responsible for the neuroprotective effect has not yet been elucidated. Using UHPLC-Q-TOF-MS plant metabolomics technology and modern activity evaluation methods, and through correlation analysis of various data, the effective ingredients of traditional Chinese medicine are clarified. Compared with traditional research methods, this research method has more comprehensive and more efficient analysis results and plays a very good role in promoting basic research on the quality of traditional Chinese medicine. It has become a new trend and hot spot in the research of active ingredients of traditional Chinese medicine in recent years [14, 15]. We used ultra-performance liquid chromatography-quadrupole time-of-flight mass spectrometry (UPLC-Q-TOF-MS), an effective technique for the systematic analysis of complex metabolomes, to analyze and identify the chemical components of *A. senticosus* from different parts of the plant [16, 17]. The MTT assay, based on colorimetry, was used to determine the H_2_O_2_-induced oxidative damage to PC12 cells. Lastly, using Pearson's correlation analysis, the differential marker results of different parts of *A. senticosus* and the anti-H_2_O_2_ oxidative damage results of the corresponding parts were correlated to find the material basis of antioxidative stress damage in different parts of *A. senticosus*.

## 2. Experimental

### 2.1. Materials

The *A. senticosus* plant samples were collected from Yichun, Heilongjiang Province, China, and were identified by Professor Zhong Guoyue of the Jiangxi University of Traditional Chinese Medicine. The roots, seeds, and leaves of the samples were collected, dried in air, ground, dried to a constant weight, and stored in a desiccator until use. As a control, 2-chloro-L-phenylalanine was purchased from Shanghai Hengbo Biotechnology Co., Ltd. (99% purity; Shanghai, China). Methanol, acetonitrile, and formic acid were purchased from Shanghai Baiye Biotechnology Center (LC-MS grade; Shanghai, China). Highly differentiated rat pheochromocytoma cells (PC12) were purchased from the Institute of Basic Medicine, Chinese Academy of Medical Sciences (Beijing, China). Thiazolyl blue tetrazolium bromide (MTT) was purchased from Beijing Mengyimei Biotechnology Co., Ltd. (Beijing, China). The purifier was purchased from Merck Millipore (D24 UV; Massachusetts, USA). The ultrasonic instrument was purchased from Shenzhen Redbond Electronics Co., Ltd. (PS-60AL; Shenzhen, China). The centrifuge was purchased from Thermo Fisher Scientific (Heraeus Fresco 17; Massachusetts, USA). The grinder was purchased from Shanghai Jingxin Technology Co., Ltd. (JXFSTPRP-24; Shanghai, China). The balance was purchased from Sartorius (BSA124S-CW; Göttingen, Germany). Other equipment includes Nexera UHPLC LC-30A Shimadzu (Kyoto, Japan); high-resolution mass spectrometer Triple TOF 5600 AB Sciex (Foster City, CA, USA); and ACQUITY UPLC BEH C18 column (1.7 *μ*m 2.1^*∗*^100 mm; Waters, USA).

### 2.2. Sample Preparation

2-Chloro-L-phenylalanine (5.03 mg) was precisely weighed, and the volume was made up with 70% methanol in a 25 mL Erlenmeyer flask. This solution was mixed and used as the mother liquor. It was diluted twentyfold (10.06 *μ*g/mL) to yield the working solution to prepare test samples.

Ten parts of *A. senticosus* roots, leaves, and seeds was extracted individually from random batches, pulverized in a grinder, and passed through a No. 3 sieve. After sieving, 1 g of the sample was weighed, fixed it to 50 mL of the working solution, and extracted using ultrasonication (250 W) for 30 min at room temperature. After resting at room temperature for 1 h, the volume was made up using the working solution and centrifuged at 12000 rpm at 4°C for 15 min. The supernatant was filtered through a 0.22 *μ*m microporous filter membrane into a 2 mL sample bottle and used as the test sample. To prepare the quality control sample (QC), all test samples were mixed together.

### 2.3. UPLC/QTOF-MS Conditions

A Shimadzu Nexera UHPLC LC-30A ultra-high-performance liquid chromatography system was used; the flow rate was set to 0.4 mL/min, and the volume of the sample injection was 5 *μ*L. The mobile phase comprised 0.1% formic acid in water (A) and acetonitrile (B). The multistep linear elution gradient program was as follows: 0–3.5 min, 95–85% A; 3.5–6 min, 85–70% A; 6–6.5 min, 70–70% A; 6.5–12 min, 70–30% A; 12–12.5 min, 30–30% A; 12.5–18 min, 30–0% A; 18–22 min, 0–0% A. A UPLC BEH C18 column (1.7 *μ*m^*∗*^2.1^*∗*^100 mm; Waters) was used.

The AB 5600 Triple TOF mass spectrometer can collect primary and secondary mass spectrometry data based on IDA (Information Dependant Acquisition) function (Analyst TF 1.7; AB Sciex). In each data collection cycle, the molecular ions with the strongest intensity and greater than 100 were selected to obtain the corresponding secondary mass spectrometry data. Bombardment energy: 40 eV, collision energy difference: 20 V, temperature: 550°C. To ensure the quality of the final collected data and method, the TOF-MS was calibrated after the analysis of every 4 samples. The relative standard deviations (RSDs) of the retention times and typical peak intensities (including internal standards) in the QC samples were used to evaluate data quality [18].

### 2.4. Data Analysis

Mass spectrometry data were collected in the positive and negative ion modes using UPLC-Q-TOF/MS, and Progenesis QI software was used to import the original mass spectra [19]. 2-Chloro-L-phenylalanine was used as an internal standard to normalize the data. Principal component analysis (PCA) and orthogonal projection potential structure discriminant analysis (OPLS-DA) were used to ensure data quality and model reliability. The default 7-fold cross-validation and 200 random permutation tests were performed using SIMCA-P to avoid overfitting of the OPLS-DA model. In the OPLS-DA model, the data with VIP scores >1 and *p* value <0.05 were selected as differential metabolites. The existing components in *A. senticosus* were used to establish an MS/MS database and perform material identification on the data.

### 2.5. Protective Effects of Different Parts of *A. senticosus* in H_2_O_2_-Induced Oxidative Damage in PC12 Cells

All samples obtained from the metabolomic processing method were filtered through a microporous filter membrane, dried under a stream of nitrogen, weighed, and reconstituted with dimethyl sulfoxide (DMSO) to a concentration of 100 mg/mL. The experiment was divided into a control group, H_2_O_2_ injury model group, and drug group. The control group and the 6 administration groups were treated with 200, 160, 120, 80, 40, and 20 *μ*g/mL, and the optimal dose was determined to be 80 *μ*g/mL. Next, a H_2_O_2_-induced oxidative damage model of PC12 cells was established. We found that when the H_2_O_2_ concentration was 400 *μ*m/L, the cell viability was closest to half of the control group. Thus, we chose 400 *μ*m/L as the concentration for studies using the oxidative damage model. After the preliminary experiment, the concentration of all samples was set to 80 *μ*g/mL, and the concentration of oxidative damage modeling was 400 *μ*m/L. PC12 cells were planted in a 96-well plate with a seed plate density of 1.25^*∗*^104/well. After 24 hours of incubation, except for the control group and the model group, the other groups were given the above-mentioned sample at a concentration of 80 *μ*g/mL in each group of cells. After continuing to incubate for 12 hours, except for the control group, the other groups added 100 *μ*L of 400 *μ*m/L H_2_O_2_ to damage PC12 cells. After 12 hours, add 10% MTT to react for 4 hours. Next, DMSO was added to dissolve the formazan crystals. Lastly, the absorbance was measured at 570 nm to determine the effect of the intervention of samples on the survival rate of PC12 cells in H_2_O_2_-induced oxidative stress.

## 3. Results

### 3.1. Multivariate Analysis of Plant Metabolism Data

A total of 130 compounds, including 93 in ESI+ mode and 37 in ESI− mode, were identified or tentatively characterized from three parts of *A. senticosus*. By matching the retention time, precise molecular mass, and fragment ions of the metabolites with the local database and combining with the literature, their structure was determined. The mass spectrum information is listed in Table S1.

**Figure 1:**
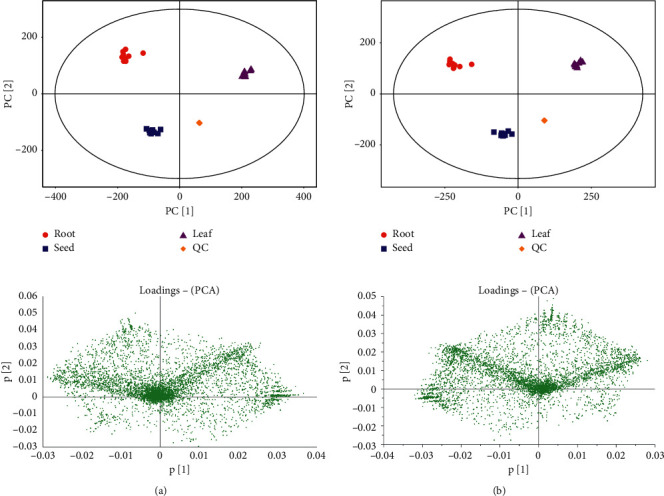
PCA score and PCA loadings ((a) positive ion mode; (b) negative ion mode).

To determine the effects of different metabolites of *A. senticosus*, the metabolic data of the three different parts of this plant were analyzed using multivariate statistical analysis. PCA is the most commonly used method in metabolomics research [20]. It shows the internal structure to make the data variables clearer, so that high-dimensional images can be converted into low-dimensional images with as little loss of spatial information as possible [21]. Sample classification information can be obtained from the graph. In the present study, the known data were analyzed using PCA and good results have been obtained. The PCA of all samples including the QC sample is represented as a scatter plot and a loading plot (Figure 1). The metabolites of the three different parts were significantly different, and the separation trend was obvious. The peak intensity and retention time of the ion peaks in the QC sample (including the internal standards) in the positive and negative ion modes had a high degree of overlap (Figures 1(a) and 1(b)). The data quality met the requirements of statistical analysis.

**Figure 2:**
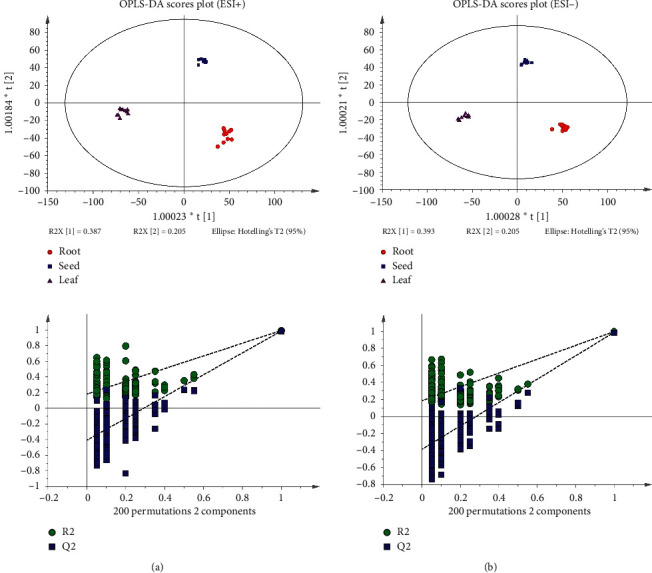
OPLS-DA and permutation score plots of the roots, seeds, and leaves.

The signal responses of the different parts of *A. senticosus* in the ESI+ and ESI− modes were combined, and their distribution was analyzed. The results showed significant differences in the metabolites between the two modes. In Figure 2, the PCA and OPLS-DA plots show that the roots, leaves, and seeds have good clustering. Pareto diagrams show that the model is not overfitted. The quality of OPLS-DA is usually evaluated based on R2Y and Q2 and in our study, and these values were determined to be 0.989 and 0.983, respectively, in the positive mode and 0.994 and 0.987, respectively, in the negative mode. These results indicated that the OPLS-DA model was reliable.

### 3.2. Identification of Different Metabolites from the Different Parts of *A. senticosus*

**Figure 3:**
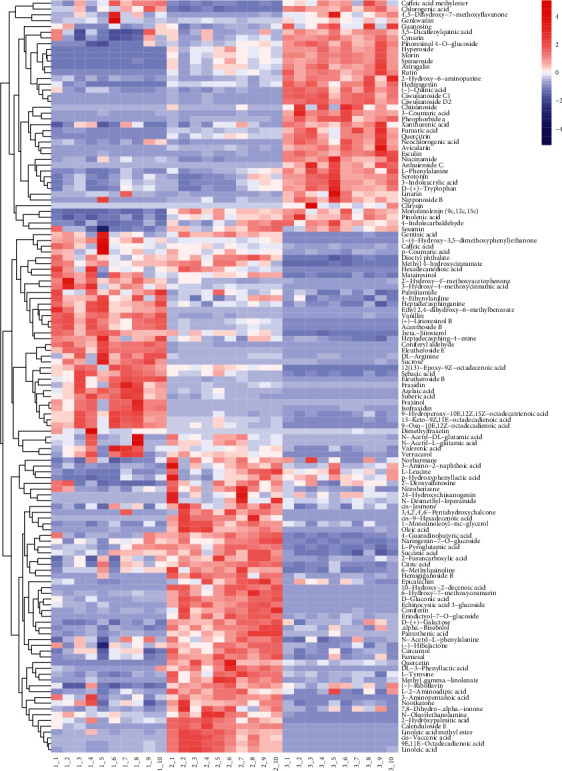
Identification and matching results of differential markers between different parts of *Acanthopanax senticosus.*

After processing the data using multivariate statistical methods, univariate statistical analysis (UVA) was used to retain the data with VIP >1 and a significance of *p* < 0.05 for the differential metabolites. Based on the literature review of the existing components of *A. senticosus*, using information from the secondary mass spectrometry database, and the application of the corresponding fragmentation law to identify the data containing the peaks corresponding to the secondary fragments, the relationship between the different components of the plant obtained from the different parts was established. The chromatograms of extracts from three different parts of *A. senticosus* showed that there were 130 different metabolites: 39 in roots, 51 in seeds, and 40 in leaves (Figures S3, Table S3). As seen in Figure 3, the accumulation of 130 metabolites is affected by the different parts and shows obvious changes in the heat map. In cluster 1, the flavonoids—including genkwanin, hyperoside, morin, rutin, quercitrin, and avicularin—and terpenoids—including hederagenin, ciwujianoside C1, ciwujianoside D2, and 3-coumaric acid—appear to be accumulated at high concentrations in the leaves. These levels were similar to the concentration of the compounds extracted from *A. senticosus* leaves [22, 23]. The lignans—namely, matairesinol, (+)-lirioresinol B, acanthoside B, and eleutheroside E—and the phenolic acids—namely, gentisic acid, caffeic acid, vanillin, and coniferyl aldehyde—in cluster 2 were present in high concentration in the roots. These findings were consistent with the reports of compounds isolated and identified from the roots of *A. senticosus* [24]. Amino acids—including L-leucine, L-2-aminoadipic acid, 3-aminopentanoic acid, and N-acetyl-L-glutamic acid—and phenolic acids—including p-hydroxyphenyllactic acid, L-tyrosine, 3-amino-2-naphthoic acid, and 2-furancarboxylic acid—were present in high concentration in the seeds in cluster 3. These phenolic acids have also been reported in previous studies [25, 26]. It is well known that plants containing high concentrations of phenols and flavonoids have various health benefits such as antioxidant effects [27, 28].

### 3.3. Effects of Different Parts of *A. senticosus* in Protecting PC12 Cells from H_2_O_2_-Induced Oxidative Stress and Correlation Analysis of Its Different Metabolites

**Figure 4:**
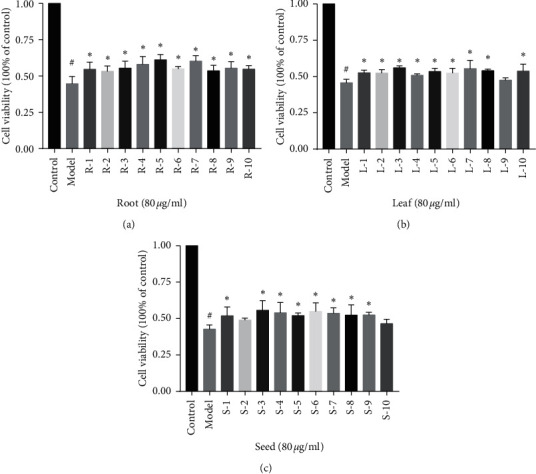
Survival rate of H_2_O_2_-induced PC12 cells after pretreatment with different parts of *Acanthopanax senticosus* (*X* ± *S n* = 3). Note: compared to the control group, ^#^*p* < 0.05; compared to the H_2_O_2_ group, ^*∗*^*p* < 0.05.

To evaluate the neuroprotective effects of the different parts of *A. senticosus*, we used a total of 30 samples from 3 different parts of the plant (roots, leaves, and seeds) to protect PC12 cells from the oxidative stress damage induced by H_2_O_2_. The results are shown in Figure 4, 10 batches of samples from roots all showed significant activity, 9 batches of samples from leaves showed significant activity, and 8 batches of samples from seeds showed significant activity.

**Figure 5:**
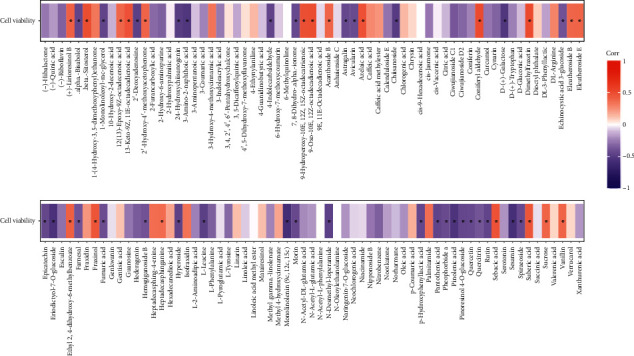
Correlation of different metabolites.

We calculated the correlation coefficient between the data of the different metabolites of the different parts of *A. senticosus* (roots, seeds, leaves) and their protective effects in H_2_O_2_-induced oxidative stress damage of the samples of that part, using Pearson's method. As shown in Figure 5, 52 compounds with significant correlations were found, of which 20 were positively correlated metabolites and 32 were negatively correlated metabolites. Using relative quantitative analysis of metabolomics, it was found that the relative content of the positively related substances in the roots was slightly higher than that in the leaves and seeds, whereas the relative content of negatively related substances in the seeds and leaves was slightly higher than that in roots. This is consistent with the findings that the root of *A. senticosus* could potentially be used in the management of neurodegenerative diseases [29].

### 3.4. Biological Significance of Related Metabolites

Our findings indicated that the relative contents of vanillin, sucrose, eleutheroside E, eleutheroside B, 2′-hydroxy-4′-methoxyacetophenone, dimethylfraxetin, and acanthoside B among others with significant positive correlation in the roots of *A. senticosus* were slightly higher than those in the leaves and seeds. Vanillin is an abundantly available and inexpensive natural product, which has significant antioxidant, anti-inflammatory, and neuroprotective effects [30, 31]. Sucrose is an energy carrier, which provides plants with the energy and carbon needed for growth and development and also improves their antioxidant activity [32, 33]. Previous studies report that eleutherosides E and B have significant antioxidant and neuroprotective effects and can delay the progression of AD [34, 35]. Acanthoside B can improve the symptoms caused by scopolamine in amnestic mice by regulating cholinergic function and restoring antioxidant status [36]. 2′-Hydroxy-4′-methoxyacetophenone is an important phenolic compound constituting Chinese herbal medicine. It can inhibit endoplasmic reticulum stress-mediated oxidative stress and improve the endothelial function in mice [37].

## 4. Conclusion

In this study, UPLC-Q/TOF-MS was used to analyze the plant metabolomics of different parts (roots, seeds, and leaves) of *A. senticosus*. Pearson's correlation analysis was used to determine the effects of the different metabolites in H_2_O_2_-induced oxidative stress in PC12 cells. Our results showed that there were 130 different metabolites in the different parts of *A. senticosus*, of which 52 were significantly related to the H_2_O_2_-induced oxidative stress in PC12 cells. Among the 52 metabolites, 20 and 32 were found to be positively and negatively correlated, respectively. The results from our study indicated that the efficacy of the roots of *A. senticosus* was slightly stronger than that of the seeds and leaves. The method of this study based on plant metabolomics can provide a detailed metabolomics overview of different parts of a plant and prove to be useful in performing an overall metabolomics analysis. The findings from our study will help better understand the metabolism of *A. senticosus*, provide important data for the study of different parts of the plant, and promote the rational development of medicinal plants.

## Data Availability

The data used to support the findings of this study are available from the corresponding author upon request.

## Supplementary Materials

Supplementary Materials Supplementary figure: TIC of all QC samples and TIC of different parts of *A. senticosus.*

## References

[1] Erkkinen M. G., Kim M. O., Geschwind M. D. (2018). Clinical neurology and epidemiology of the major neurodegenerative diseases. *Cold Spring Harbor Perspectives in Biology*.

[2] Sun B.-L., Li W.-W., Zhu C. (2018). Clinical research on alzheimer’s disease: progress and perspectives. *Neuroscience Bulletin*.

[3] Pizzino G., Irrera N., Cucinotta M. (2017). Oxidative stress: harms and benefits for human health. *Oxidative Medicine and Cellular Longevity*.

[4] Kaminskyy V. O., Zhivotovsky B. (2014). Free radicals in cross talk between autophagy and apoptosis. *Antioxidants & Redox Signaling*.

[5] Bettegazzi B., Pelizzoni I., Salerno Scarzella F. (2019). Upregulation of peroxiredoxin 3 protects afg3l2-KO cortical neurons in vitro from oxidative stress: a paradigm for neuronal cell survival under neurodegenerative conditions. *Oxidative Medicine and Cellular Longevity*.

[6] Pelizzoni I., Macco R., Morini M. F., Zacchetti D., Grohovaz F., Codazzi F. (2011). Iron handling in hippocampal neurons: activity-dependent iron entry and mitochondria-mediated neurotoxicity. *Aging Cell*.

[7] Musgrove R. E., Helwig M., Bae E.-J. (2019). Oxidative stress in vagal neurons promotes parkinsonian pathology and intercellular *α*-synuclein transfer. *Journal of Clinical Investigation*.

[8] Wiatrak B., Kubis-Kubiak A., Piwowar A., Barg E. (2020). PC12 cell line: cell types, coating of culture vessels, differentiation and other culture conditions. *Cells*.

[9] Zhao Y., Wang Q., Wang Y., Li J., Lu G., Liu Z. (2019). Glutamine protects against oxidative stress injury through inhibiting the activation of PI3K/Akt signaling pathway in parkinsonian cell model. *Environmental Health and Preventive Medicine*.

[10] Wang Y., Liu S., Wang R., Shi L., Liu Z., Liu Z. (2020). Study on the therapeutic material basis and effect of Acanthopanax senticosus (Rupr. et Maxim.) Harms leaves in the treatment of ischemic stroke by PK-PD analysis based on online microdialysis-LC-MS/MS method. *Food & Function*.

[11] Zhou Y., Cheng C., Barenenko D., Wang J., Li Y., Lu W. (2018). Effects of *Acanthopanax senticosus* on brain injury induced by simulated spatial radiation in mouse model based on pharmacokinetics and comparative proteomics. *International Journal of Molecular Sciences*.

[12] Liu S.-M., Li X.-Z., Huo Y., Lu F. (2012). Protective effect of extract of Acanthopanax senticosus harms on dopaminergic neurons in Parkinson’s disease mice. *Phytomedicine*.

[13] Xie Y., Zhang B., Zhang Y. (2015). Protective effects of Acanthopanax polysaccharides on cerebral ischemia-reperfusion injury and its mechanisms. *International Journal of Biological Macromolecules*.

[14] Han L., Wang P., Wang Y. (2019). Rapid discovery of the potential toxic compounds in polygonum multiflorum by UHPLC/Q-Orbitrap-MS-Based metabolomics and correlation analysis. *Frontiers in Pharmacology*.

[15] Sun W., Chen Z., Hong J., Shi J. (2020). Promoting human nutrition and health through plant metabolomics: current status and challenges. *Biology (Basel)*.

[16] Huilan Y., Junjun D., Zhen H., Chengxin P., Shilei L., Yu X. (2015). Synthesis and analysis of phosphorylated nonapeptide adducts by LC/Q-TOF MS. *Phosphorus, Sulfur, and Silicon and the Related Elements*.

[17] Noh E., Yoon C.-Y., Lee J. H. (2016). A liquid chromatography-quadrupole-time of flight mass spectrometry (LC-Q-TOF MS) study for analyzing 35 corticosteroid compounds: elucidation of MS/MS fragmentation pathways. *Bulletin of the Korean Chemical Society*.

[18] Wang D., Wang Q., Chen R., Yang S., Li Z., Feng Y. (2019). Exploring the effects of Gastrodia elata Blume on the treatment of cerebral ischemia-reperfusion injury using UPLC-Q/TOF-MS-based plasma metabolomics. *Food & Function*.

[19] Zhang J., Yang W., Li S. (2016). An intelligentized strategy for endogenous small molecules characterization and quality evaluation of earthworm from two geographic origins by ultra-high performance HILIC/QTOF MSE and Progenesis QI. *Analytical and Bioanalytical Chemistry*.

[20] Bai Z. Z., Hu X. J., Tian J. P., Chen P., Luo H., Huang D. (2020). Rapid and nondestructive detection of sorghum adulteration using optimization algorithms and hyperspectral imaging. *Food Chemistry*.

[21] Landgraf A. J., Lee Y. (2020). Dimensionality reduction for binary data through the projection of natural parameters. *Journal of Multivariate Analysis*.

[22] Zhang Y., Zhang A., Zhang Y. (2016). Application of ultra-performance liquid chromatography with time-of-flight mass spectrometry for the rapid analysis of constituents and metabolites from the extracts of Acanthopanax senticosus harms leaf. *Pharmacognosy Magazine*.

[23] Cheng H. C., Wei W. F., Huo J. H., Sun G. D., Wang W. M. (2016). [Identification of chemical constituents of the leaves from Acanthopanax senticosus by UPLC-Q-TOF-MS/MS]. *Zhong Yao Cai*.

[24] Shi X., Yang Y., Ren H. (2020). Identification of multiple components in deep eutectic solvent extract of Acanthopanax senticosus root by ultra-high-performance liquid chromatography with quadrupole orbitrap mass spectrometry. *Phytochemistry Letters*.

[25] Wu K. X., Liu J., Liu Y. (2018). A comparative metabolomics analysis reveals the tissue-specific phenolic profiling in two Acanthopanax species. *Molecules*.

[26] Muszynska B., Lojewski M., Rojowski J., Opoka W., Sulkowska-Ziaja K. (2015). Natural products of relevance in the prevention and supportive treatment of depression. *Psychiatria Polska*.

[27] Rice-Evans C. A., Miller N. J., Paganga G. (1996). Structure-antioxidant activity relationships of flavonoids and phenolic acids. *Free Radical Biology and Medicine*.

[28] Cho M., Lee H.-S., Kang I.-J., Won M.-H., You S. (2011). Antioxidant properties of extract and fractions from Enteromorpha prolifera, a type of green seaweed. *Food Chemistry*.

[29] He Y., Wang Y., Zhang X. (2020). Chemical characterization of small-molecule inhibitors of monoamine oxidase B synthesized from the Acanthopanax senticosus root with affinity ultrafiltration mass spectrometry. *Rapid Communications in Mass Spectrometry*.

[30] Younis N. N., Elsherbiny N. M., Shaheen M. A., Elseweidy M. M. (2020). Modulation of NADPH oxidase and Nrf2/HO-1 pathway by vanillin in cisplatin-induced nephrotoxicity in rats. *Journal of Pharmacy and Pharmacology*.

[31] Chen H., Zheng J., Ma J. (2019). Vanillin ameliorates changes in HIF-1*α* expression and neuronal apoptosis in a rat model of spinal cord injury. *Restorative Neurology and Neuroscience*.

[32] Hu L., Chen L., Liu L., Lou Y., Amombo E., Fu J. (2015). Metabolic acclimation of source and sink tissues to salinity stress in bermudagrass (Cynodon dactylon). *Physiologia Plantarum*.

[33] Sim U., Sung J., Lee H., Heo H., Jeong H. S., Lee J. (2020). Effect of calcium chloride and sucrose on the composition of bioactive compounds and antioxidant activities in buckwheat sprouts. *Food Chemistry*.

[34] Wang S., Yang X. (2020). Eleutheroside E decreases oxidative stress and NF-*κ*B activation and reprograms the metabolic response against hypoxia-reoxygenation injury in H9c2 cells. *International Immunopharmacology*.

[35] Huang D., Hu Z., Yu Z. (2013). Eleutheroside B or E enhances learning and memory in experimentally aged rats. *Neural Regeneration Research*.

[36] Karthivashan G., Kweon M.-H., Park S.-Y. (2019). Cognitive-enhancing and ameliorative effects of acanthoside B in a scopolamine-induced amnesic mouse model through regulation of oxidative/inflammatory/cholinergic systems and activation of the TrkB/CREB/BDNF pathway. *Food and Chemical Toxicology*.

[37] Choy K. W., Lau Y. S., Murugan D., Mustafa M. R. (2017). Chronic treatment with paeonol improves endothelial function in mice through inhibition of endoplasmic reticulum stress-mediated oxidative stress. *PLoS One*.

