# Supplementary material for: Different Metabolites in the Roots, Seeds, and Leaves of Acanthopanax senticosus and Their Role in Alleviating Oxidative Stress
Source: J Anal Methods Chem. 2021 Apr 15;2021:6628880. doi: 10.1155/2021/6628880 (PMC8064801; doi:10.1155/2021/6628880)
Supplement: Supplementary Materials — Supplementary figure: TIC of all QC samples and TIC of different parts of A. senticosus. [file 6628880.f1.zip › 6628880.f1/Figure Fs POS-TOTAL.pdf]

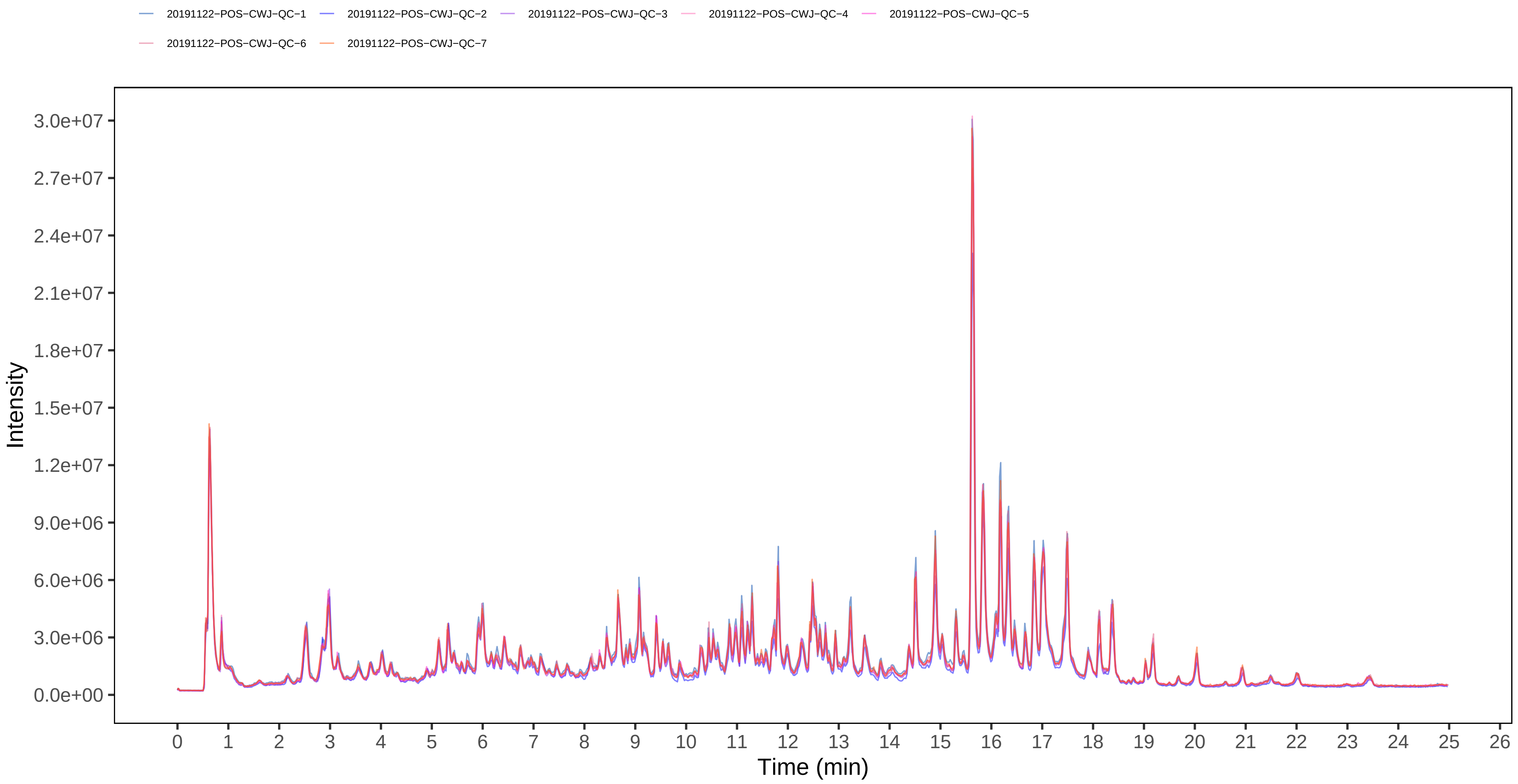
20191122−POS−CWJ−QC−1
20191122−POS−CWJ−QC−2
20191122−POS−CWJ−QC−3
20191122−POS−CWJ−QC−4
20191122−POS−CWJ−QC−5
20191122−POS−CWJ−QC−6
20191122−POS−CWJ−QC−7
Intensity
3.0e+07
2.7e+07
2.4e+07
2.1e+07
1.8e+07
1.5e+07
1.2e+07
9.0e+06
6.0e+06
3.0e+06
0.0e+00
0 1 2 3 4 5 6 7 8 9 10 11 12 13 14 15 16 17 18 19 20 21 22 23 24 25 26
Time (min)

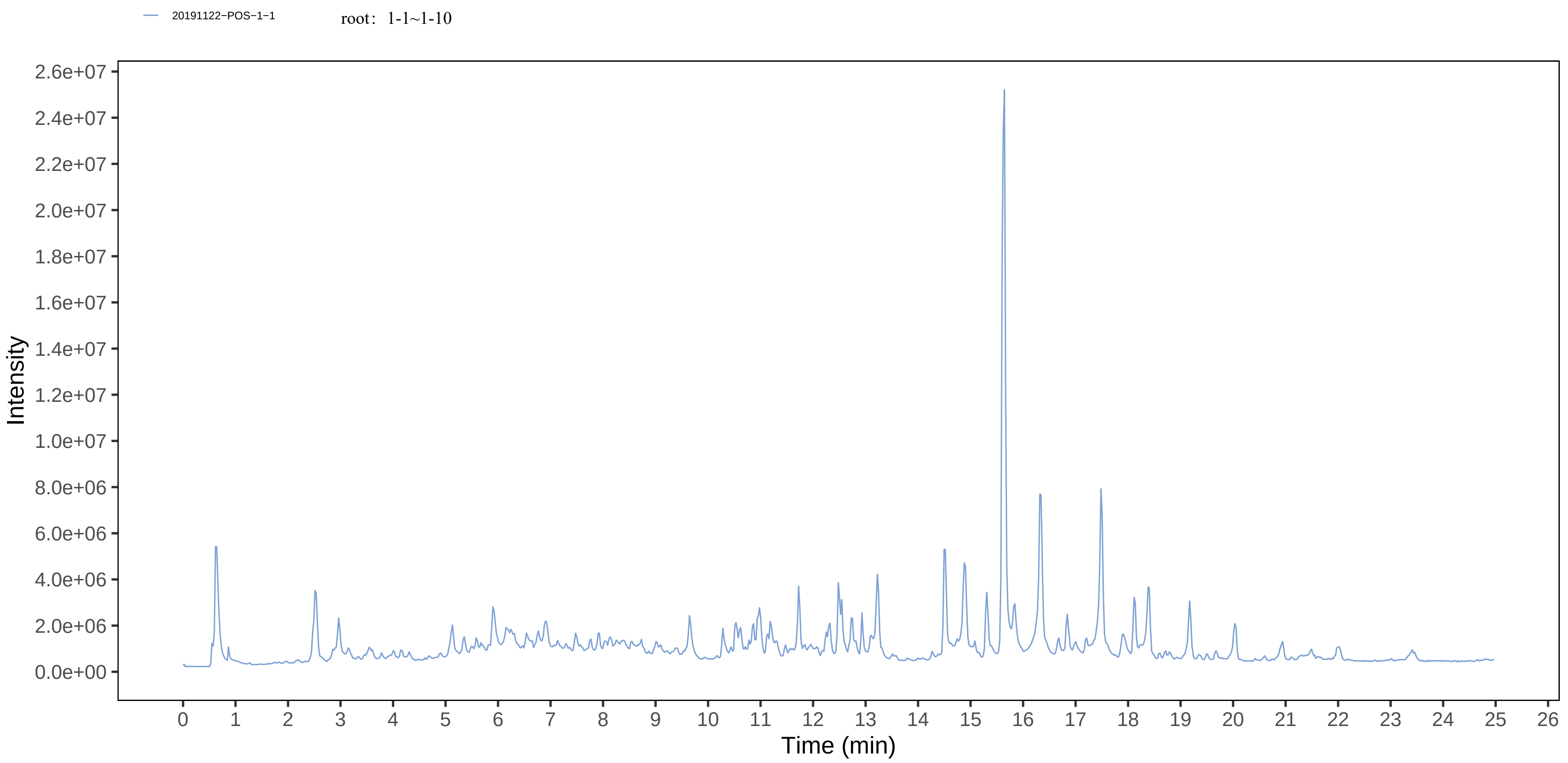

20191122−POS−1−1
root：1-1~1-10
Intensity
Time (min)

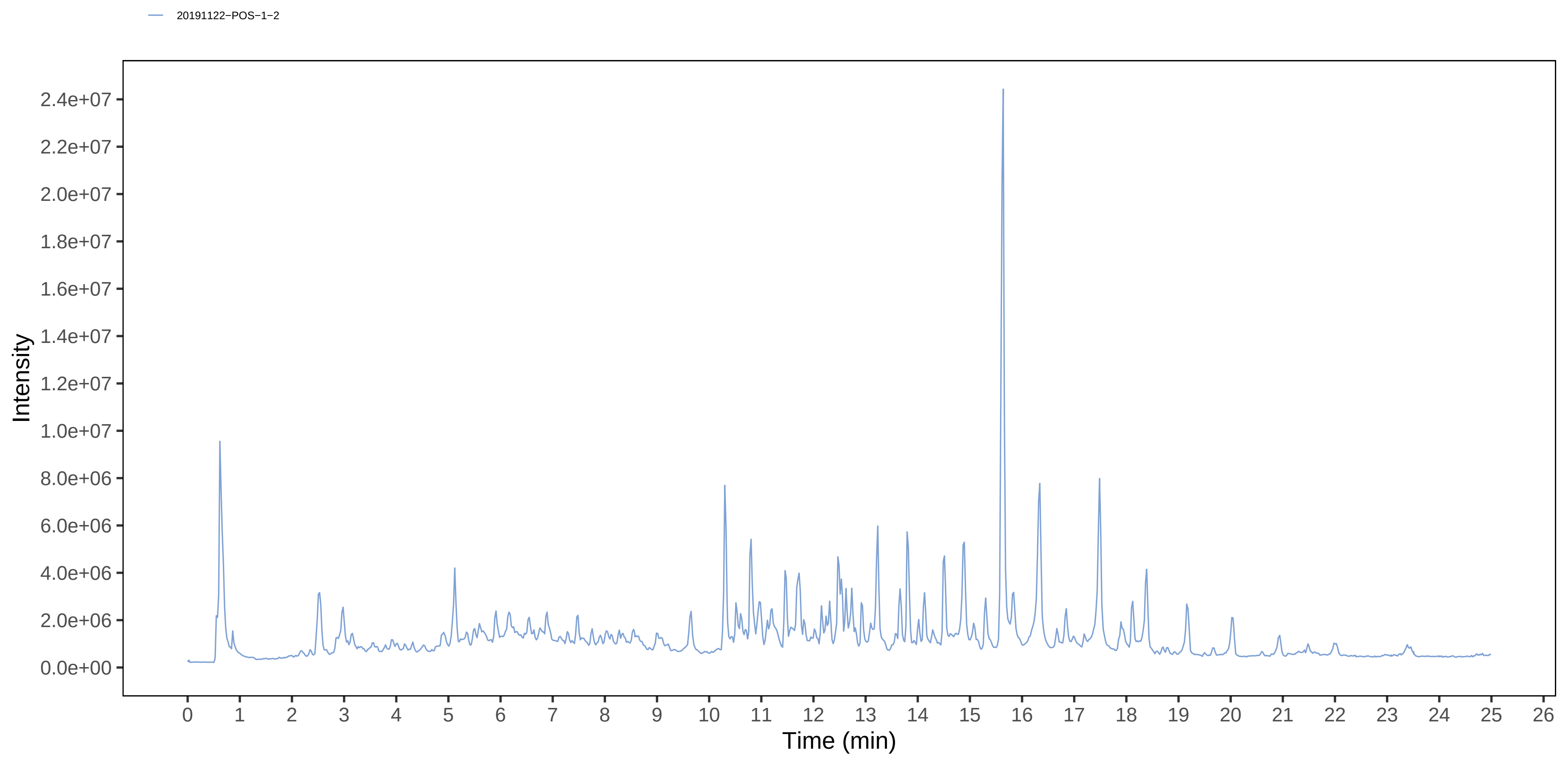

20191122−POS−1−2
Intensity
2.4e+07
2.2e+07
2.0e+07
1.8e+07
1.6e+07
1.4e+07
1.2e+07
1.0e+07
8.0e+06
6.0e+06
4.0e+06
2.0e+06
0.0e+00
0 1 2 3 4 5 6 7 8 9 10 11 12 13 14 15 16 17 18 19 20 21 22 23 24 25 26
Time (min)

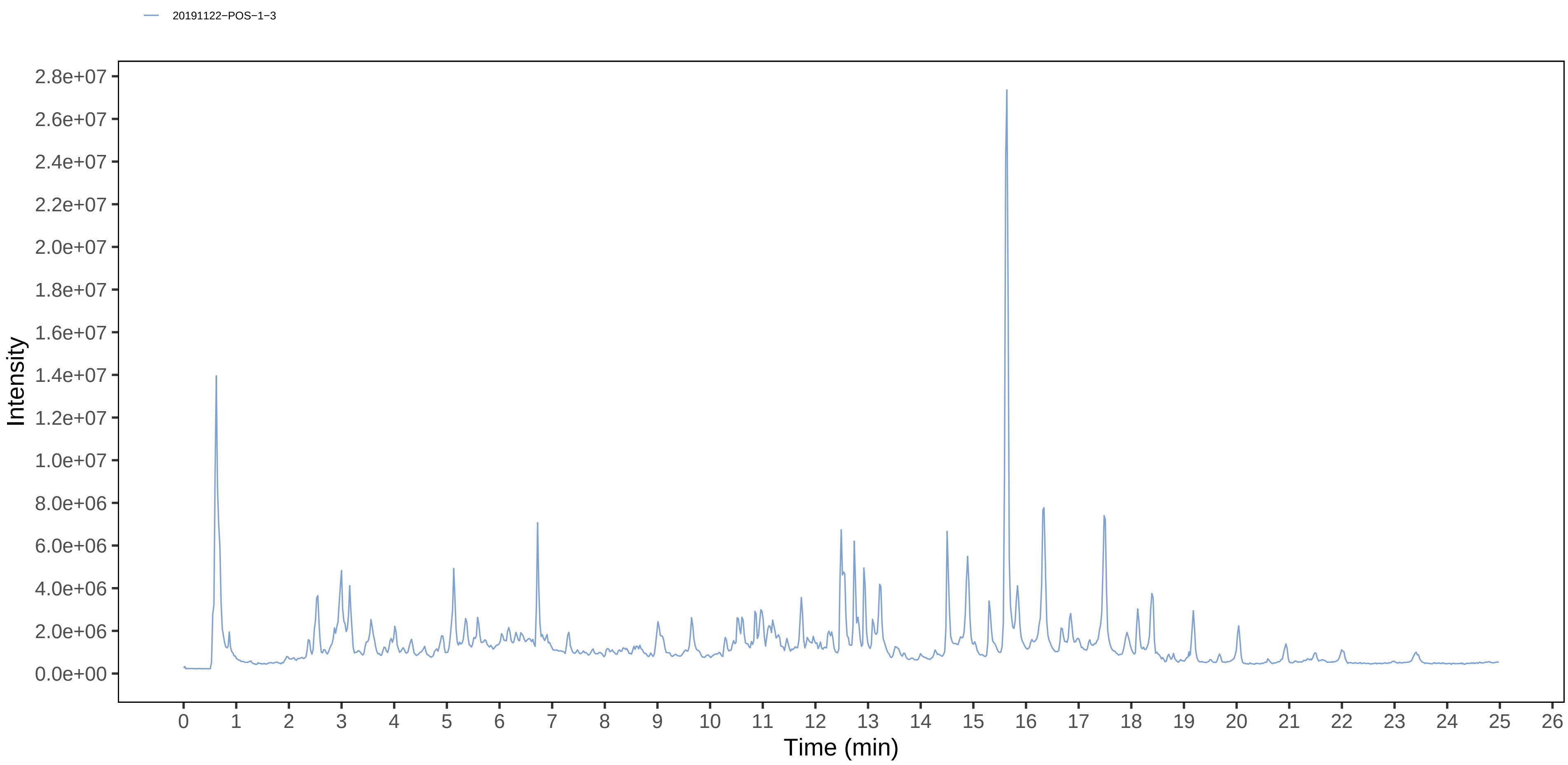

20191122−POS−1−3
Intensity
2.8e+07
2.6e+07
2.4e+07
2.2e+07
2.0e+07
1.8e+07
1.6e+07
1.4e+07
1.2e+07
1.0e+07
8.0e+06
6.0e+06
4.0e+06
2.0e+06
0.0e+00
0 1 2 3 4 5 6 7 8 9 10 11 12 13 14 15 16 17 18 19 20 21 22 23 24 25 26
Time (min)

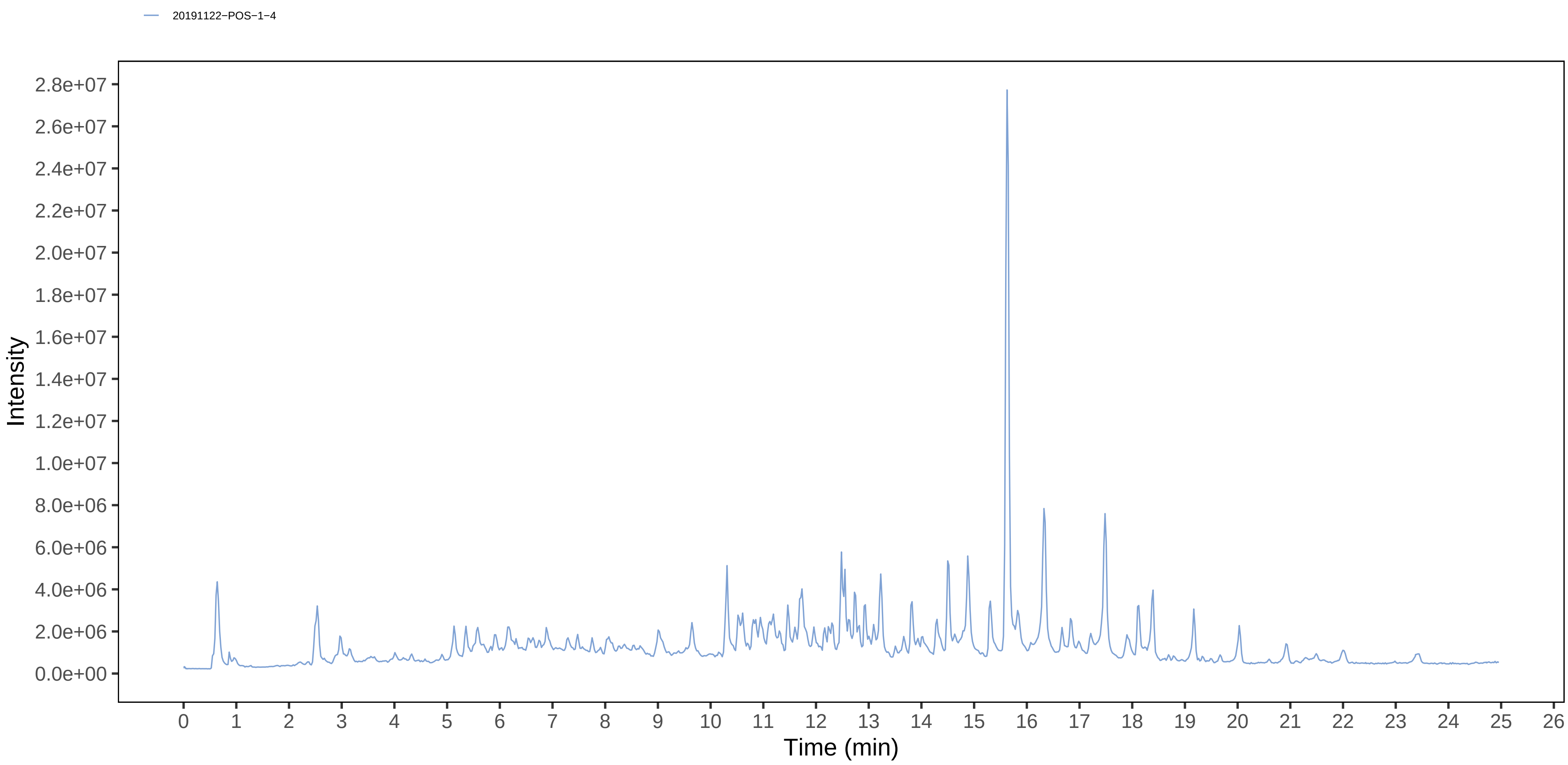

20191122−POS−1−4
Intensity
0.0e+00
2.0e+06
4.0e+06
6.0e+06
8.0e+06
1.0e+07
1.2e+07
1.4e+07
1.6e+07
1.8e+07
2.0e+07
2.2e+07
2.4e+07
2.6e+07
2.8e+07
0 1 2 3 4 5 6 7 8 9 10 11 12 13 14 15 16 17 18 19 20 21 22 23 24 25 26
Time (min)

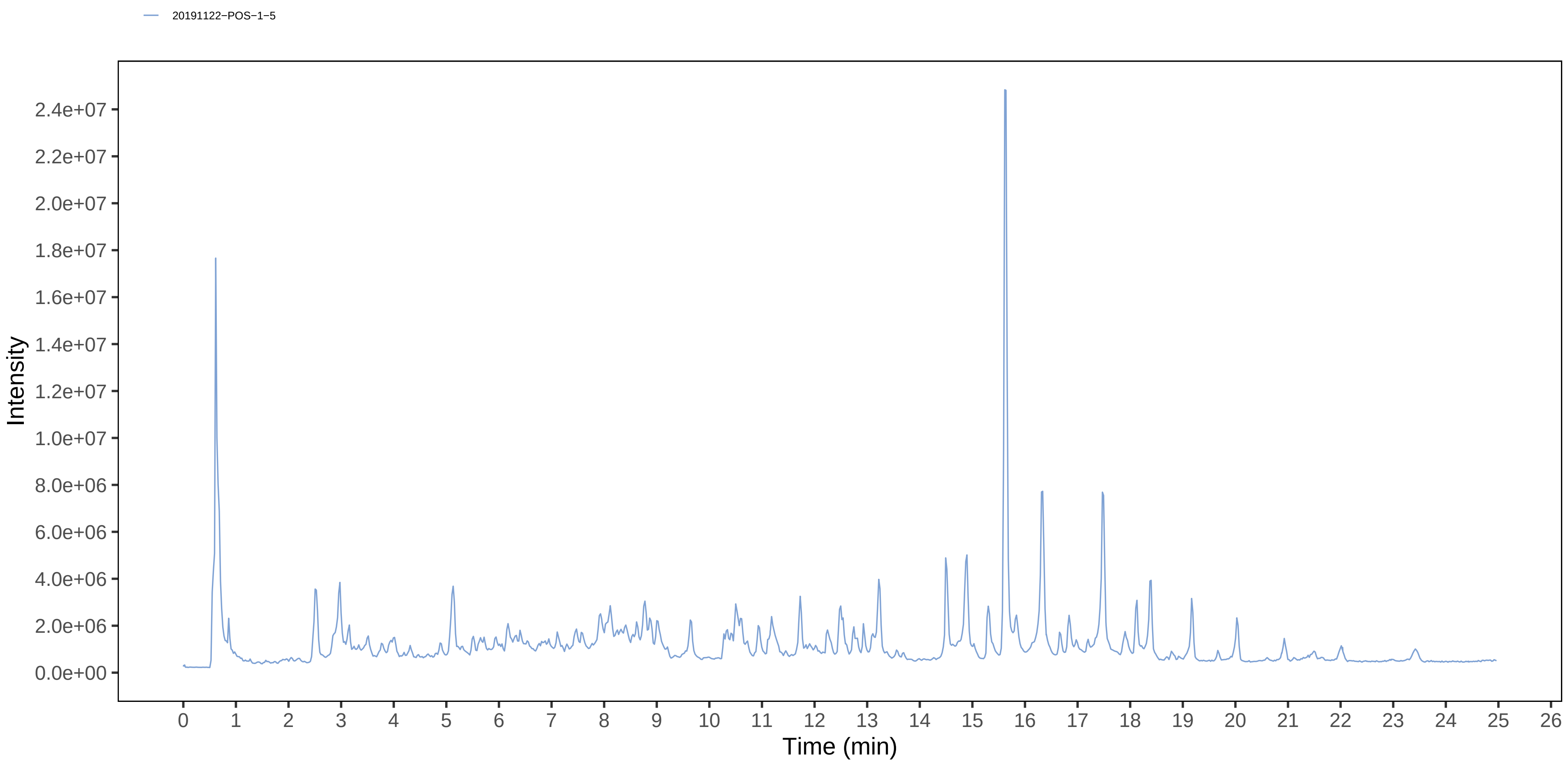

20191122−POS−1−5
Intensity
2.4e+07
2.2e+07
2.0e+07
1.8e+07
1.6e+07
1.4e+07
1.2e+07
1.0e+07
8.0e+06
6.0e+06
4.0e+06
2.0e+06
0.0e+00
0 1 2 3 4 5 6 7 8 9 10 11 12 13 14 15 16 17 18 19 20 21 22 23 24 25 26
Time (min)

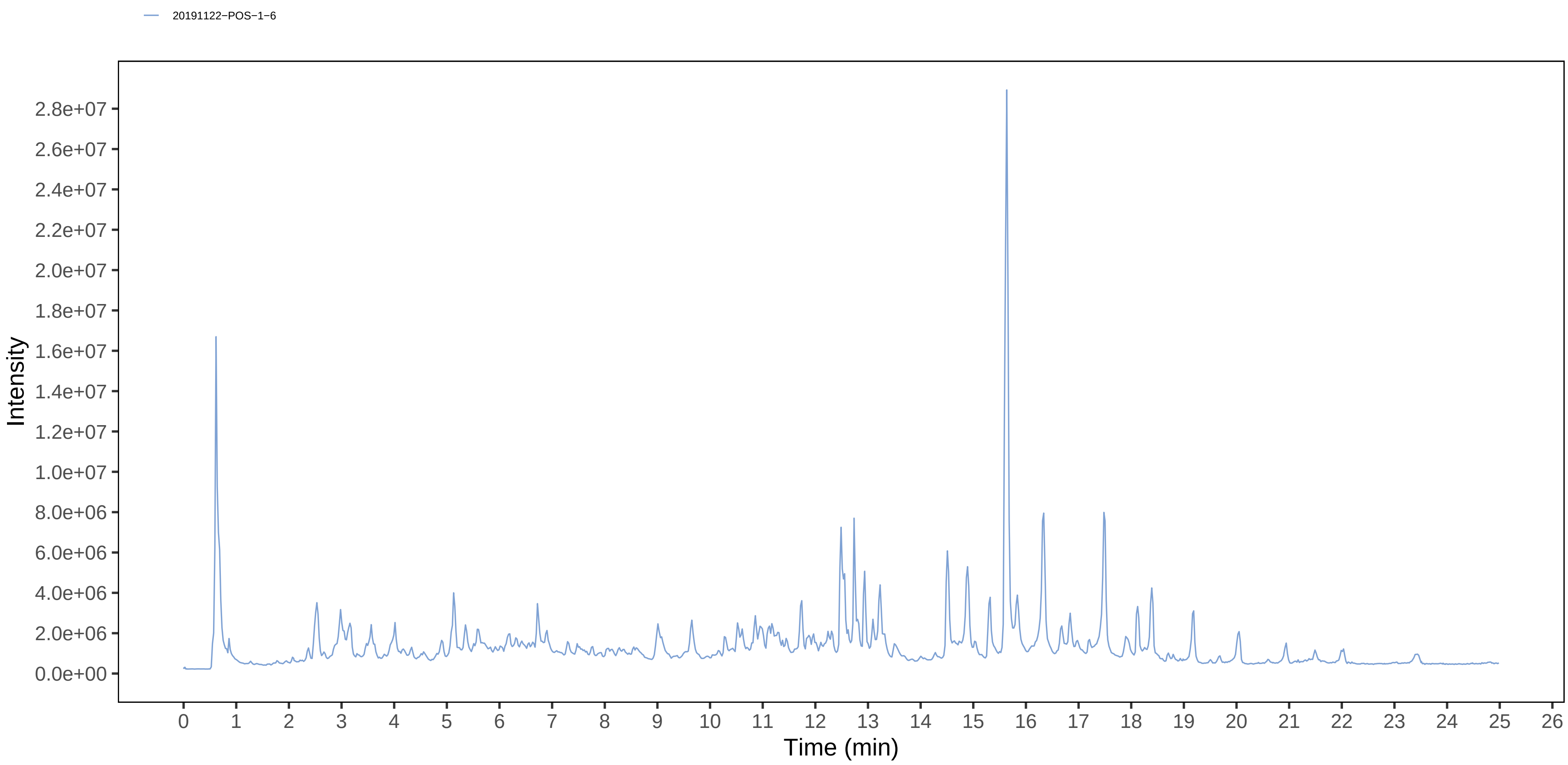
20191122−POS−1−6
Intensity
2.8e+07
2.6e+07
2.4e+07
2.2e+07
2.0e+07
1.8e+07
1.6e+07
1.4e+07
1.2e+07
1.0e+07
8.0e+06
6.0e+06
4.0e+06
2.0e+06
0.0e+00
0 1 2 3 4 5 6 7 8 9 10 11 12 13 14 15 16 17 18 19 20 21 22 23 24 25 26
Time (min)

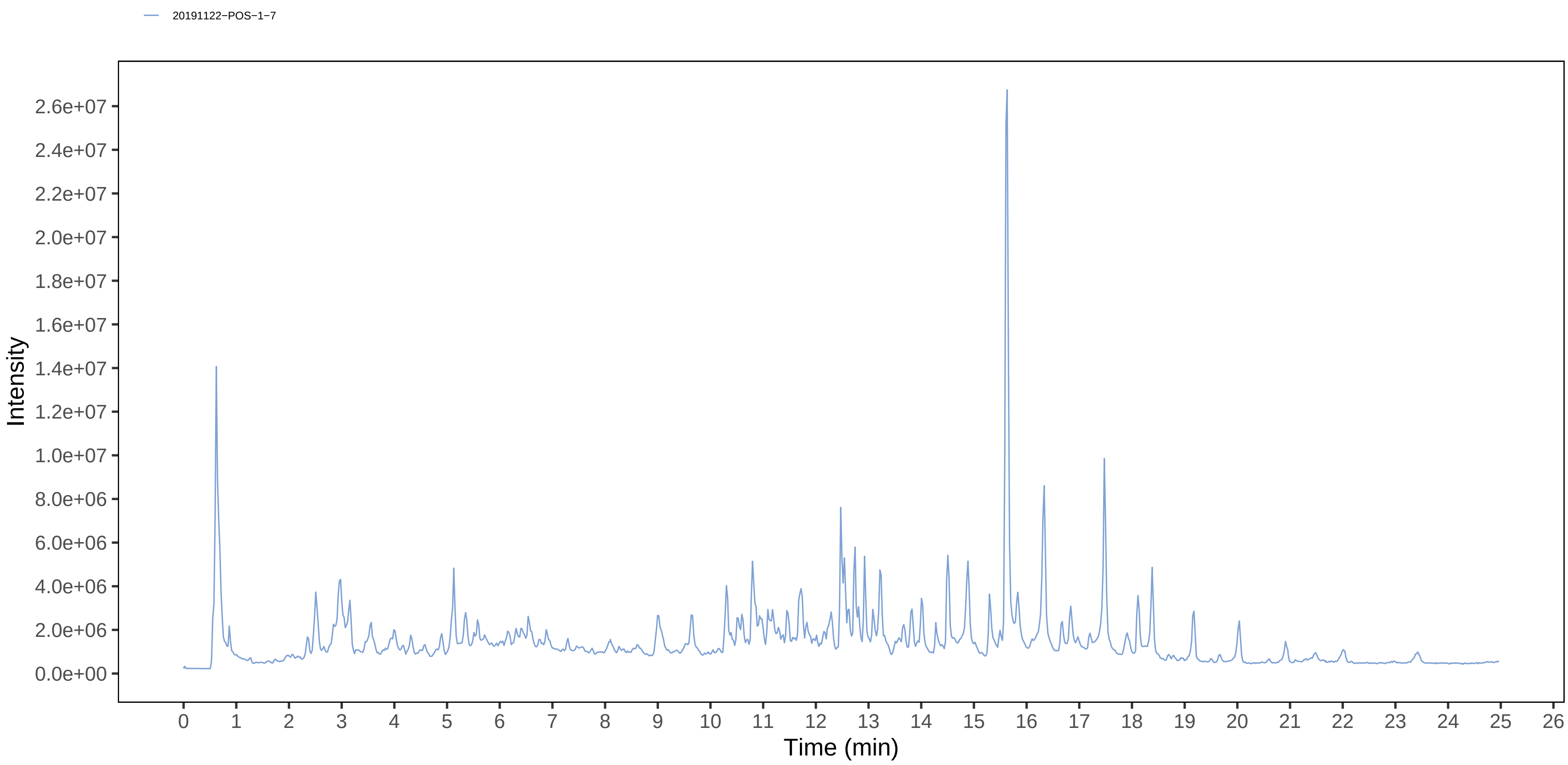
20191122−POS−1−7
Intensity
2.6e+07
2.4e+07
2.2e+07
2.0e+07
1.8e+07
1.6e+07
1.4e+07
1.2e+07
1.0e+07
8.0e+06
6.0e+06
4.0e+06
2.0e+06
0.0e+00
0 1 2 3 4 5 6 7 8 9 10 11 12 13 14 15 16 17 18 19 20 21 22 23 24 25 26
Time (min)

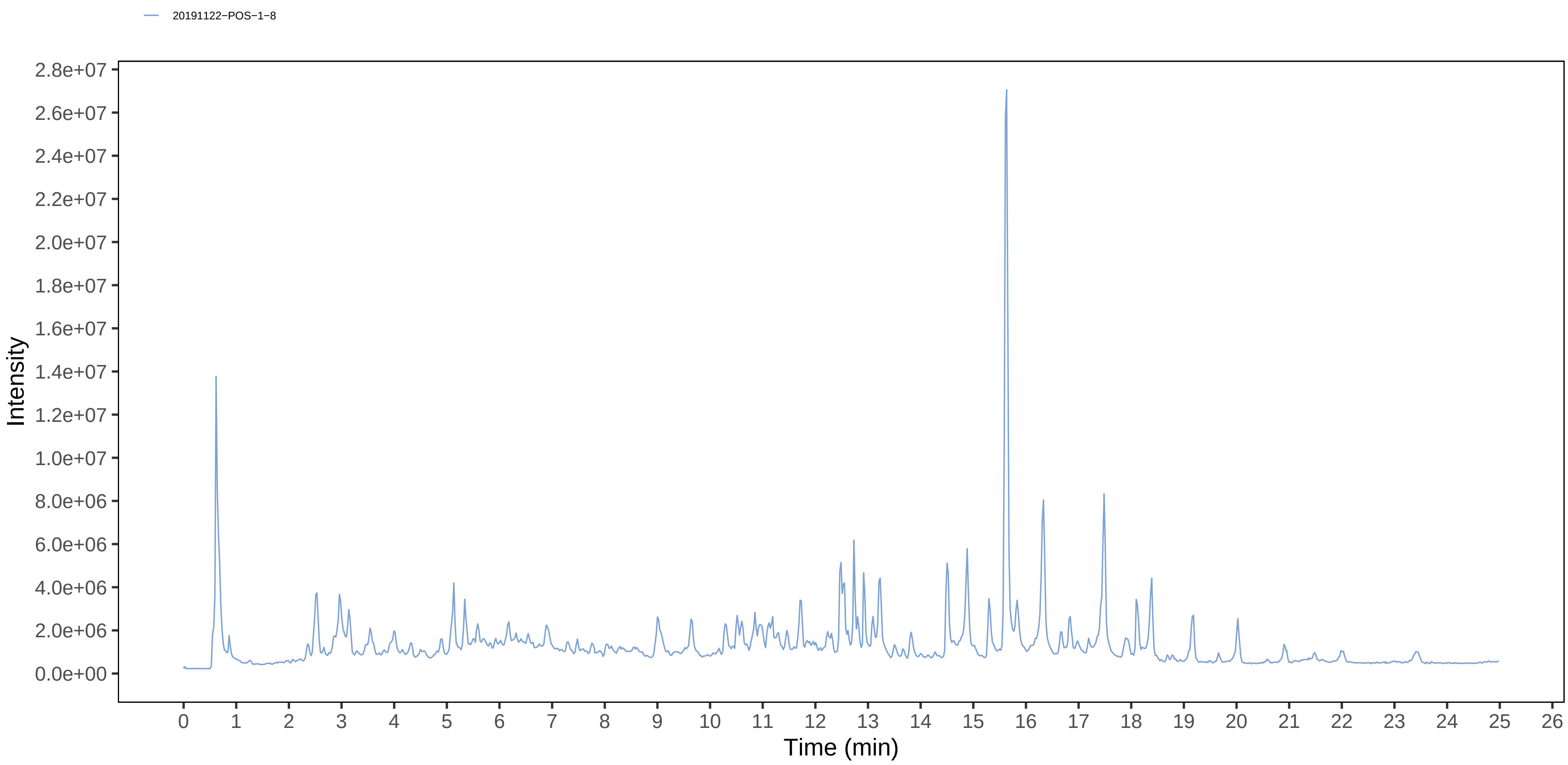

20191122−POS−1−8
Intensity
2.8e+07
2.6e+07
2.4e+07
2.2e+07
2.0e+07
1.8e+07
1.6e+07
1.4e+07
1.2e+07
1.0e+07
8.0e+06
6.0e+06
4.0e+06
2.0e+06
0.0e+00
0 1 2 3 4 5 6 7 8 9 10 11 12 13 14 15 16 17 18 19 20 21 22 23 24 25 26
Time (min)

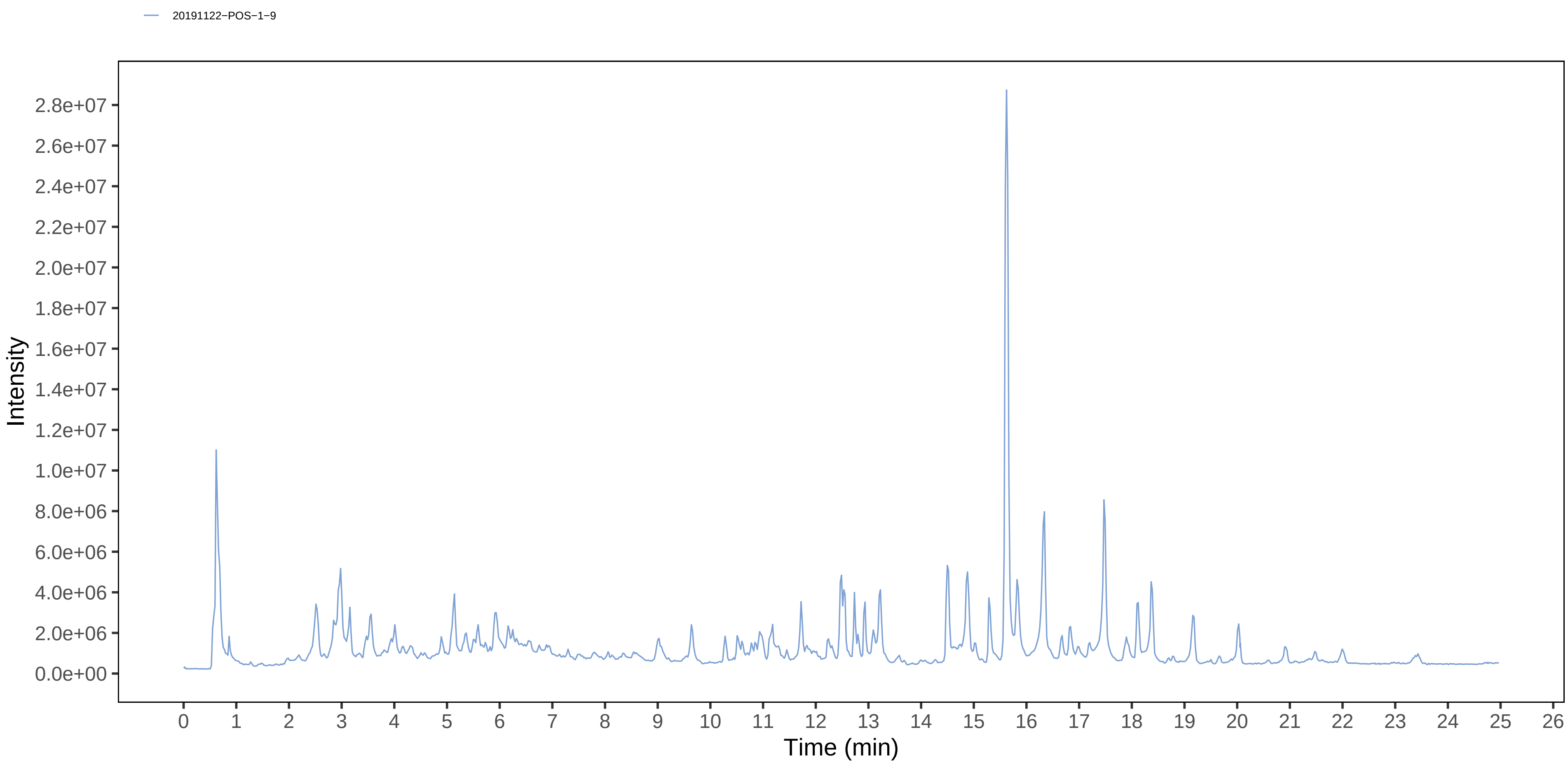

20191122−POS−1−9
Intensity
0.0e+00
2.0e+06
4.0e+06
6.0e+06
8.0e+06
1.0e+07
1.2e+07
1.4e+07
1.6e+07
1.8e+07
2.0e+07
2.2e+07
2.4e+07
2.6e+07
2.8e+07
0 1 2 3 4 5 6 7 8 9 10 11 12 13 14 15 16 17 18 19 20 21 22 23 24 25 26
Time (min)

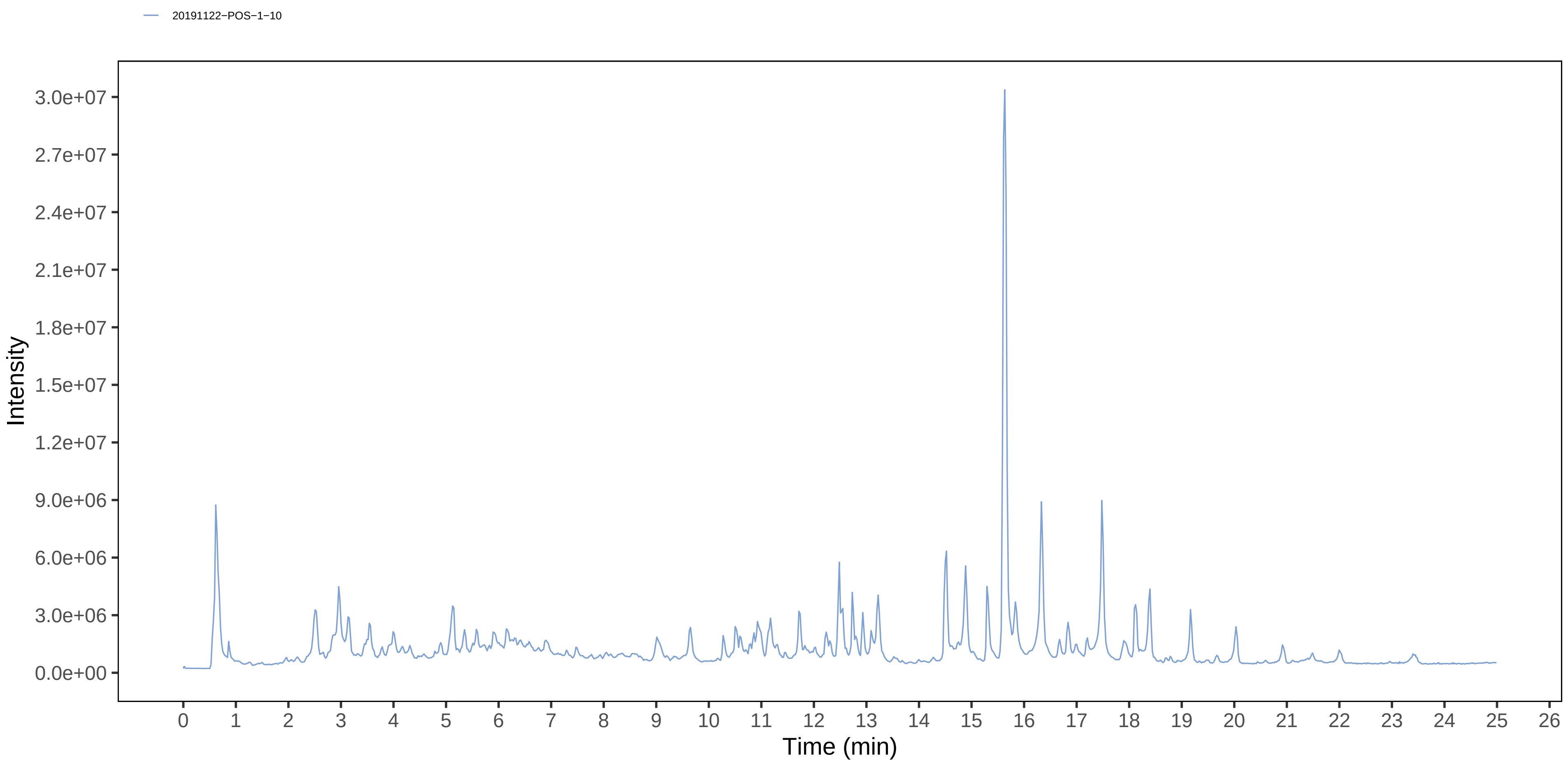

20191122−POS−1−10
Intensity
3.0e+07
2.7e+07
2.4e+07
2.1e+07
1.8e+07
1.5e+07
1.2e+07
9.0e+06
6.0e+06
3.0e+06
0.0e+00
0 1 2 3 4 5 6 7 8 9 10 11 12 13 14 15 16 17 18 19 20 21 22 23 24 25 26
Time (min)

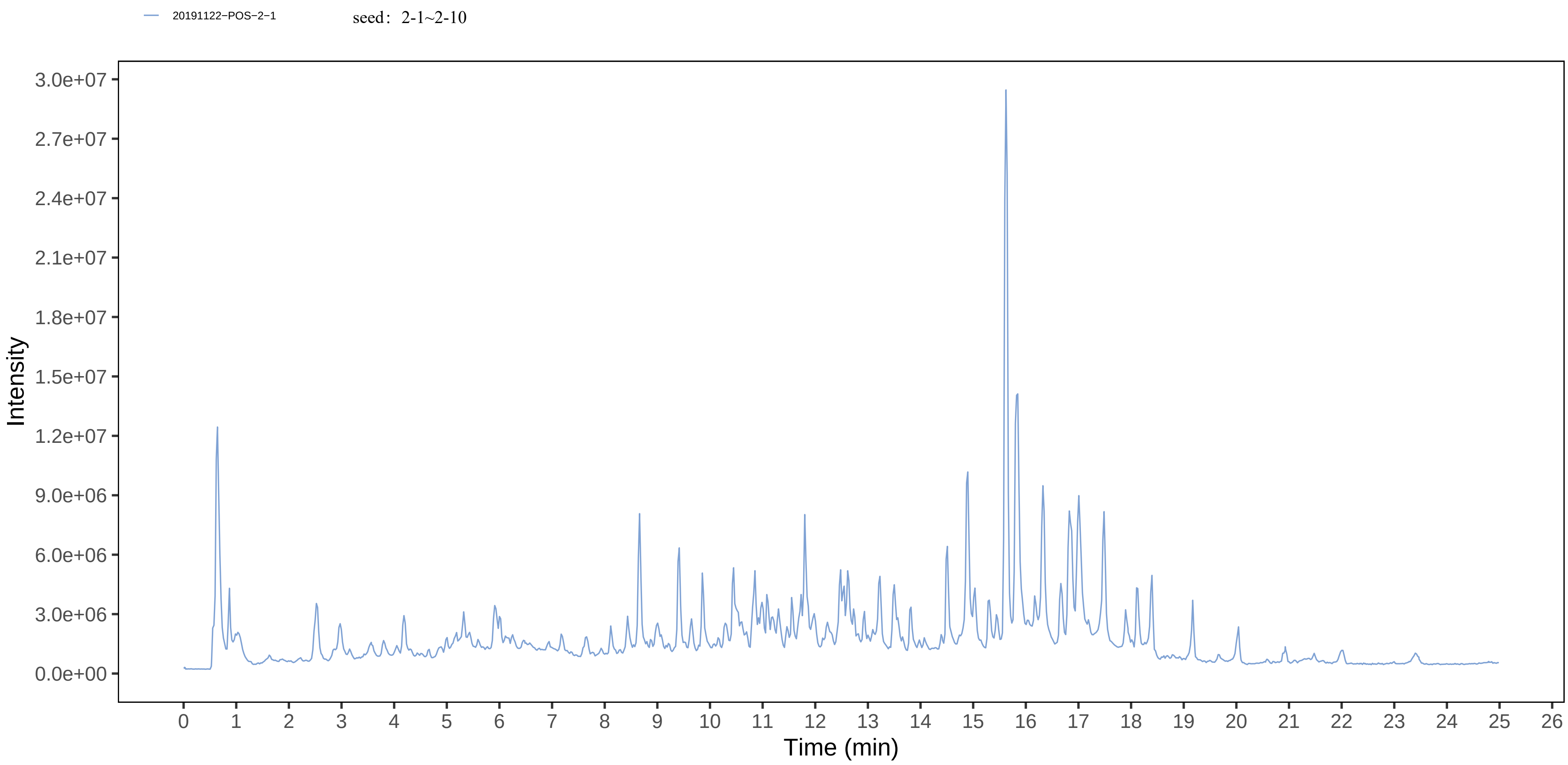

20191122−POS−2−1
seed：2-1~2-10
Intensity
3.0e+07
2.7e+07
2.4e+07
2.1e+07
1.8e+07
1.5e+07
1.2e+07
9.0e+06
6.0e+06
3.0e+06
0.0e+00
0 1 2 3 4 5 6 7 8 9 10 11 12 13 14 15 16 17 18 19 20 21 22 23 24 25 26
Time (min)

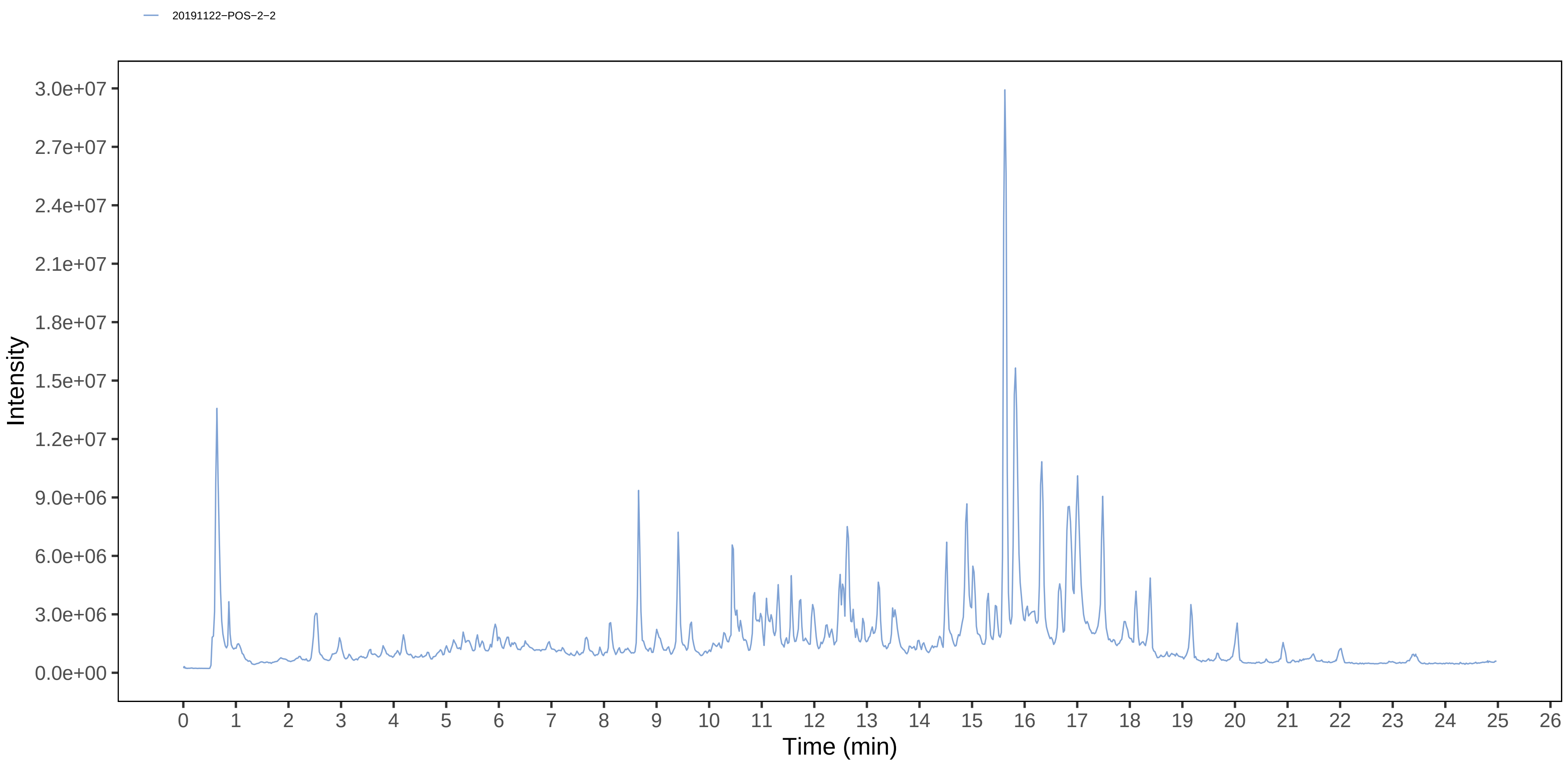

20191122−POS−2−2
Intensity
3.0e+07
2.7e+07
2.4e+07
2.1e+07
1.8e+07
1.5e+07
1.2e+07
9.0e+06
6.0e+06
3.0e+06
0.0e+00
0 1 2 3 4 5 6 7 8 9 10 11 12 13 14 15 16 17 18 19 20 21 22 23 24 25 26
Time (min)

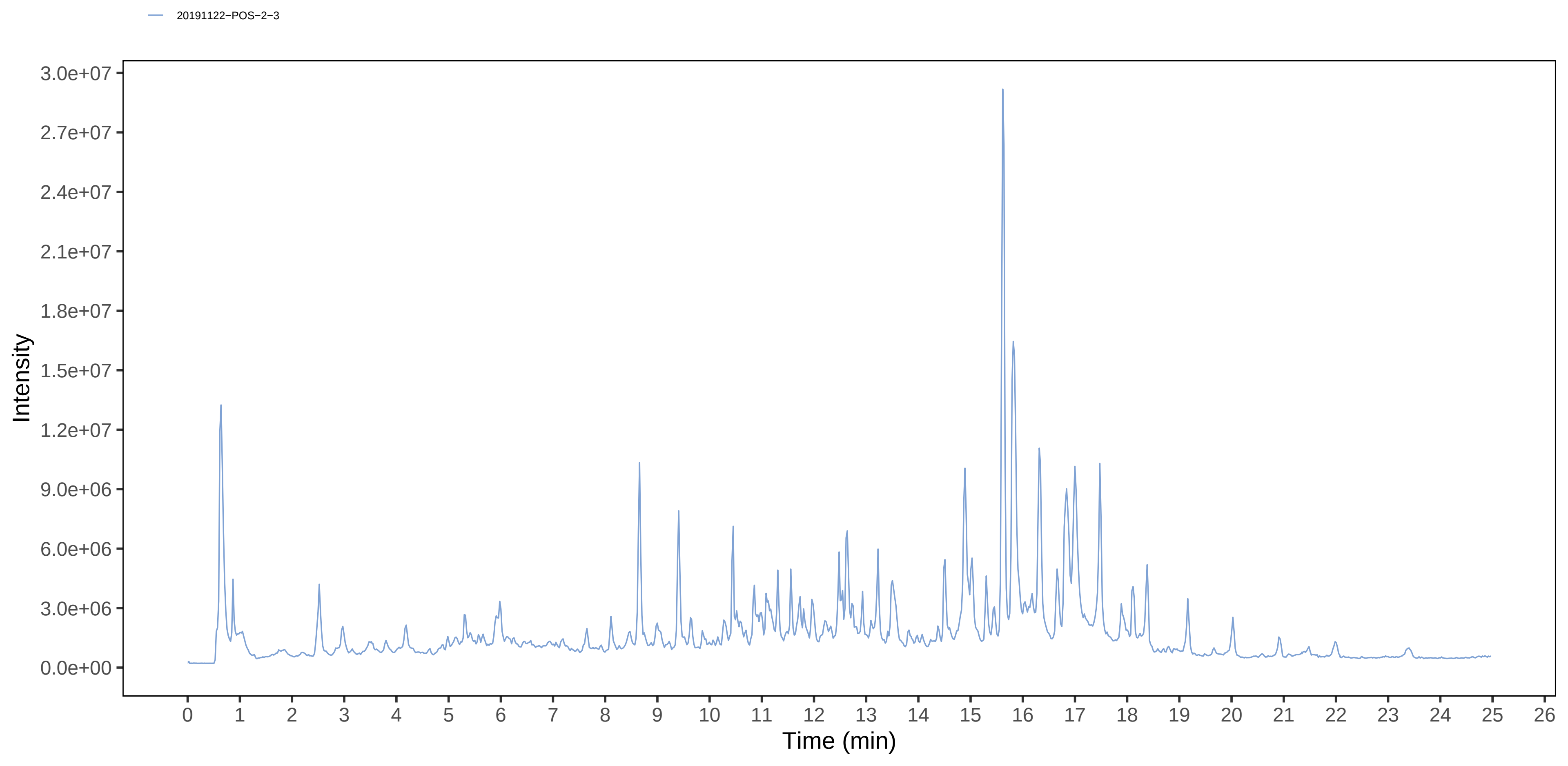
20191122−POS−2−3
Intensity
3.0e+07
2.7e+07
2.4e+07
2.1e+07
1.8e+07
1.5e+07
1.2e+07
9.0e+06
6.0e+06
3.0e+06
0.0e+00
0 1 2 3 4 5 6 7 8 9 10 11 12 13 14 15 16 17 18 19 20 21 22 23 24 25 26
Time (min)

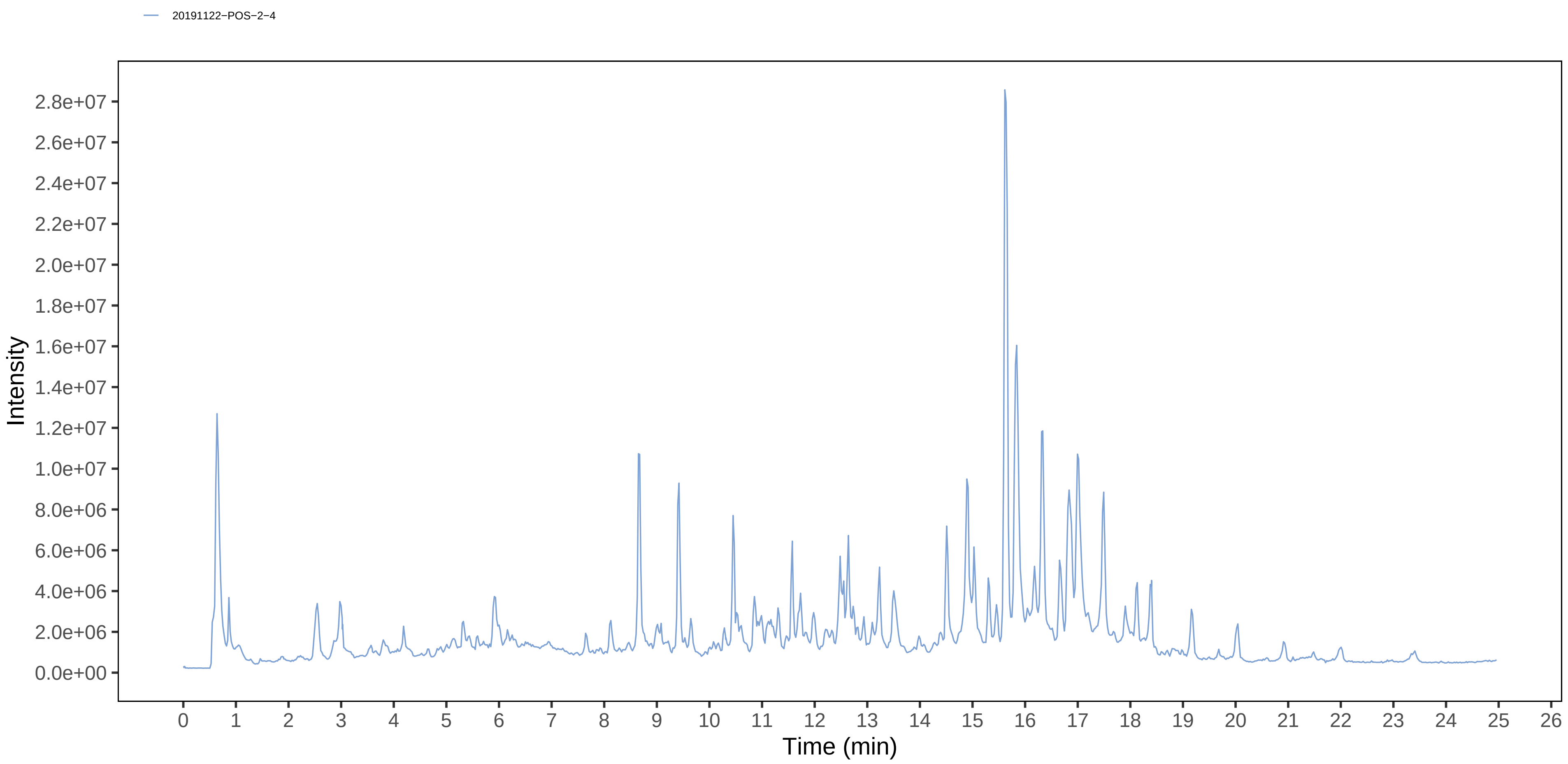
20191122−POS−2−4
Intensity
Time (min)

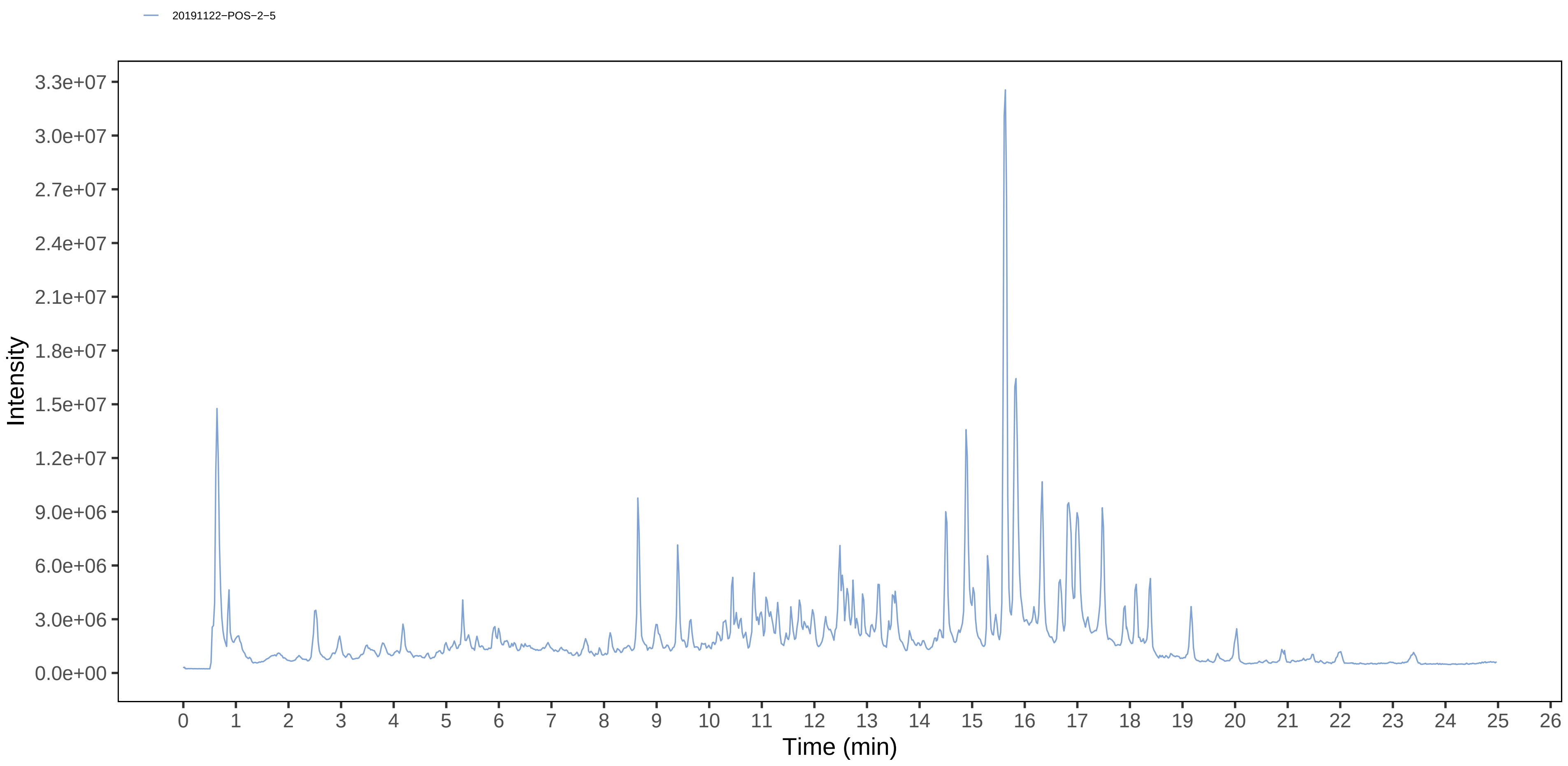

20191122−POS−2−5
Intensity
3.3e+07
3.0e+07
2.7e+07
2.4e+07
2.1e+07
1.8e+07
1.5e+07
1.2e+07
9.0e+06
6.0e+06
3.0e+06
0.0e+00
0 1 2 3 4 5 6 7 8 9 10 11 12 13 14 15 16 17 18 19 20 21 22 23 24 25 26
Time (min)

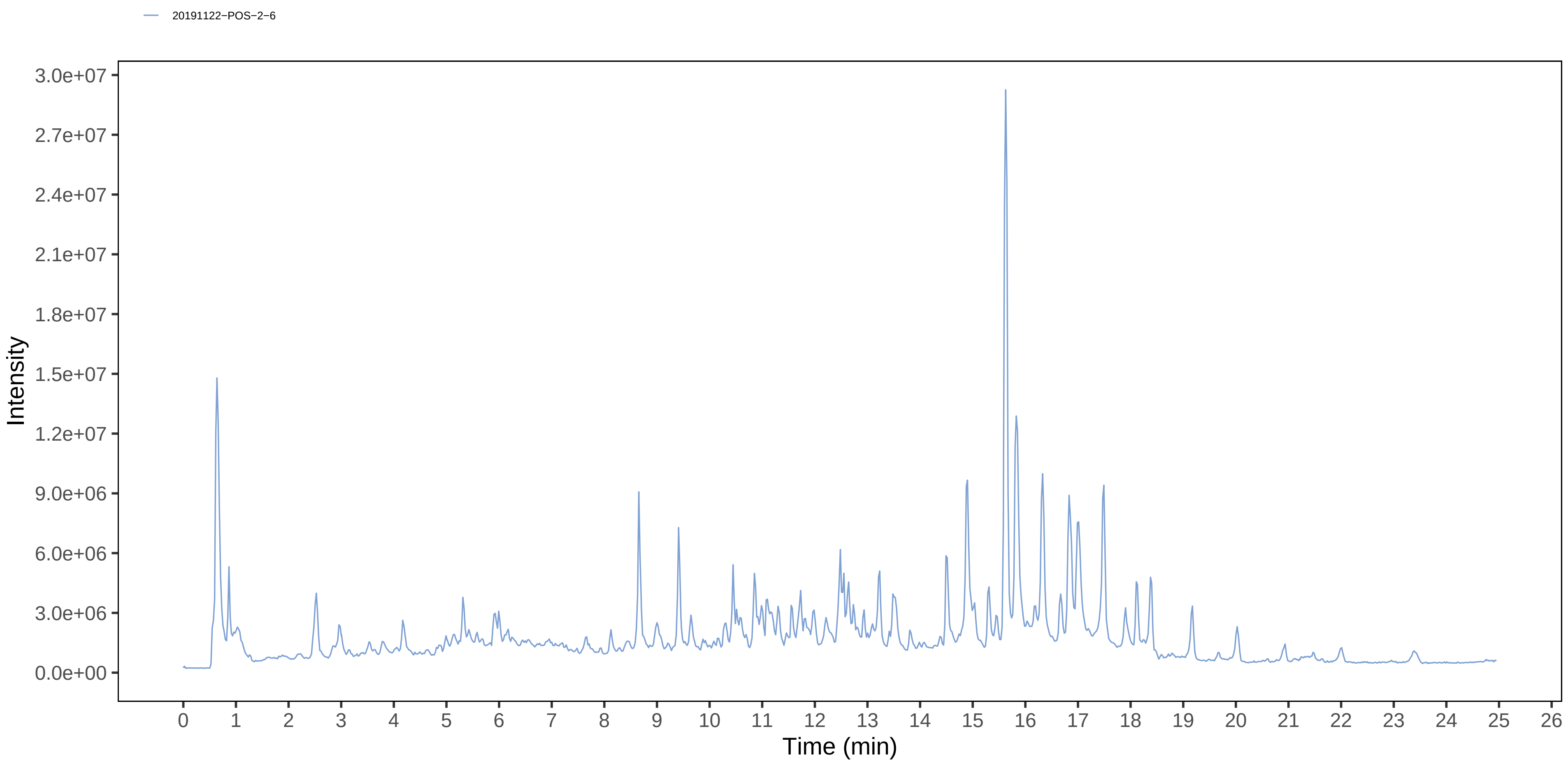

20191122−POS−2−6
Intensity
3.0e+07
2.7e+07
2.4e+07
2.1e+07
1.8e+07
1.5e+07
1.2e+07
9.0e+06
6.0e+06
3.0e+06
0.0e+00
0
1
2
3
4
5
6
7
8
9
10
11
12
13
14
15
16
17
18
19
20
21
22
23
24
25
26
Time (min)

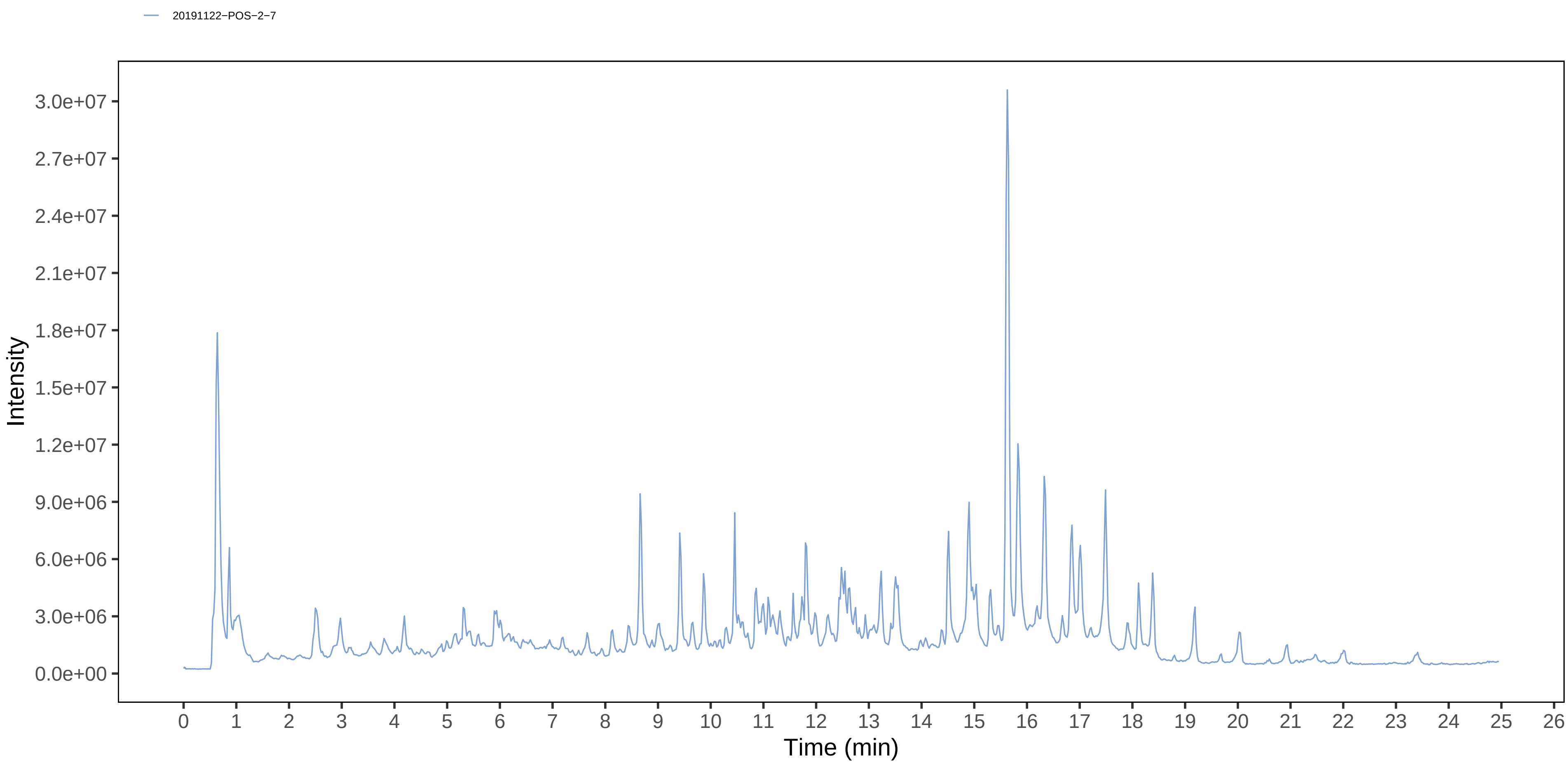

20191122−POS−2−7
Intensity
3.0e+07
2.7e+07
2.4e+07
2.1e+07
1.8e+07
1.5e+07
1.2e+07
9.0e+06
6.0e+06
3.0e+06
0.0e+00
0 1 2 3 4 5 6 7 8 9 10 11 12 13 14 15 16 17 18 19 20 21 22 23 24 25 26
Time (min)

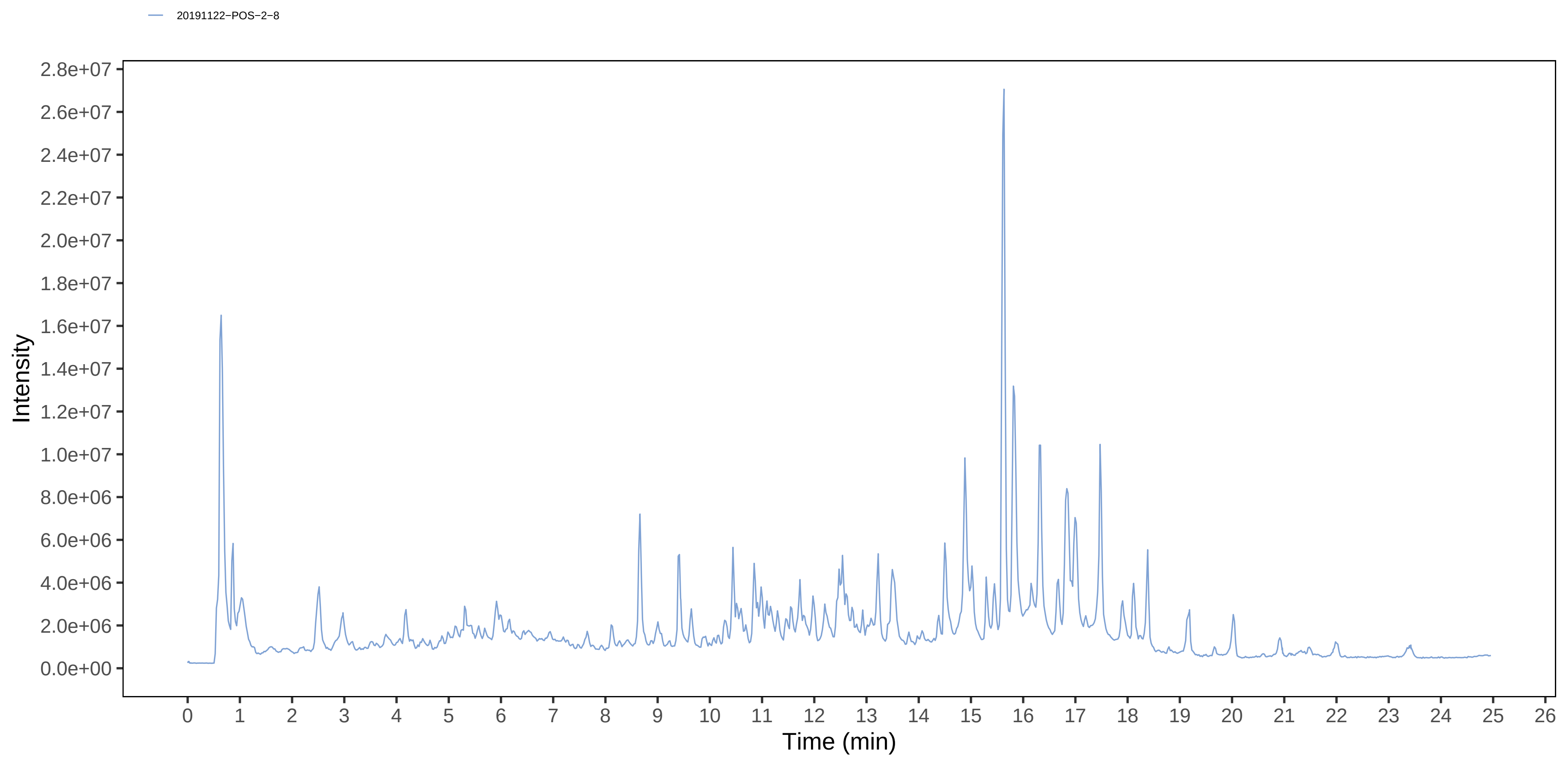

20191122−POS−2−8
2.8e+07
2.6e+07
2.4e+07
2.2e+07
2.0e+07
1.8e+07
1.6e+07
1.4e+07
1.2e+07
1.0e+07
8.0e+06
6.0e+06
4.0e+06
2.0e+06
0.0e+00
Intensity
0 1 2 3 4 5 6 7 8 9 10 11 12 13 14 15 16 17 18 19 20 21 22 23 24 25 26
Time (min)

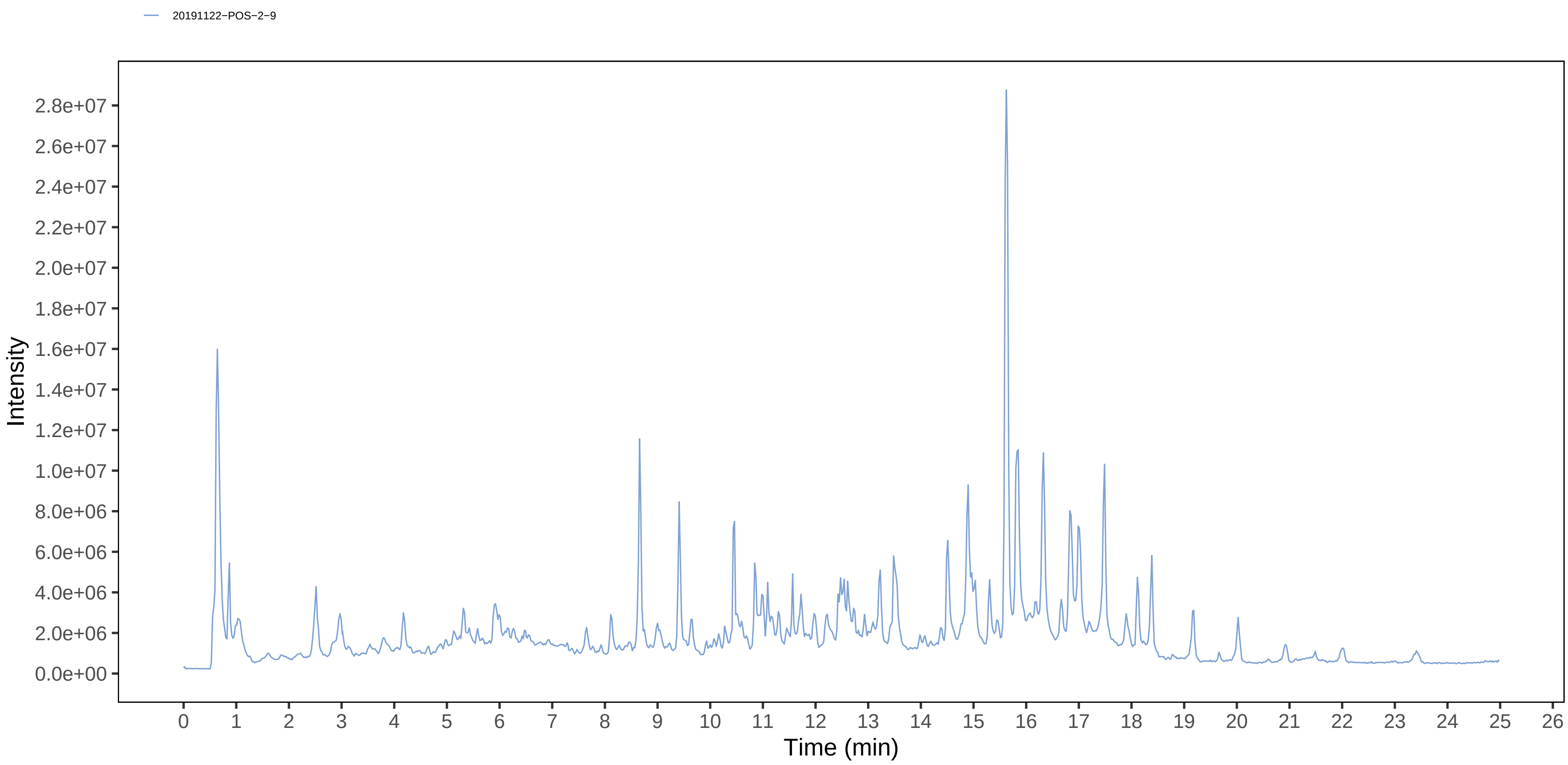
20191122−POS−2−9
Intensity
2.8e+07
2.6e+07
2.4e+07
2.2e+07
2.0e+07
1.8e+07
1.6e+07
1.4e+07
1.2e+07
1.0e+07
8.0e+06
6.0e+06
4.0e+06
2.0e+06
0.0e+00
0 1 2 3 4 5 6 7 8 9 10 11 12 13 14 15 16 17 18 19 20 21 22 23 24 25 26
Time (min)

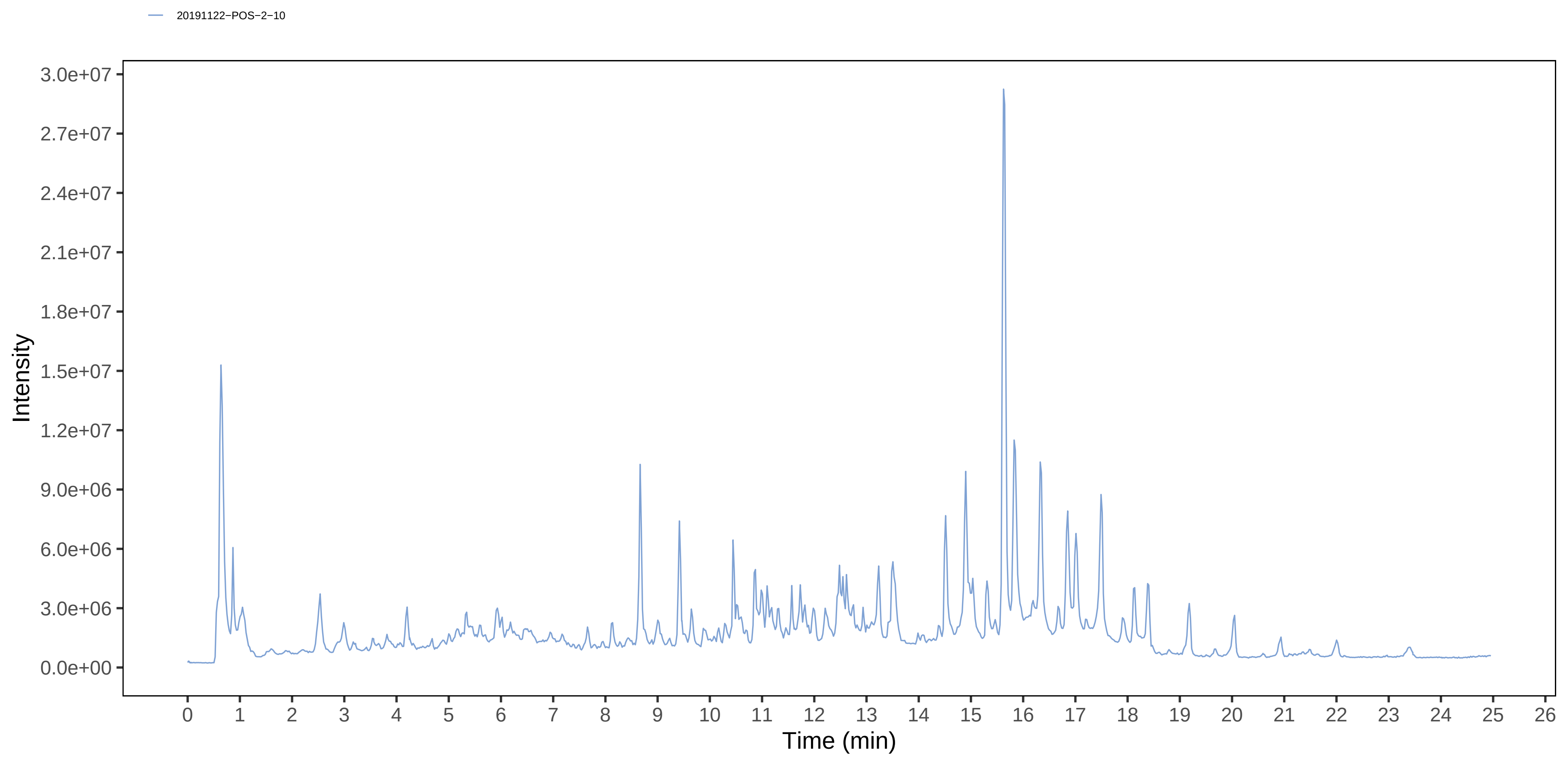
20191122−POS−2−10
Intensity
3.0e+07
2.7e+07
2.4e+07
2.1e+07
1.8e+07
1.5e+07
1.2e+07
9.0e+06
6.0e+06
3.0e+06
0.0e+00
0 1 2 3 4 5 6 7 8 9 10 11 12 13 14 15 16 17 18 19 20 21 22 23 24 25 26
Time (min)

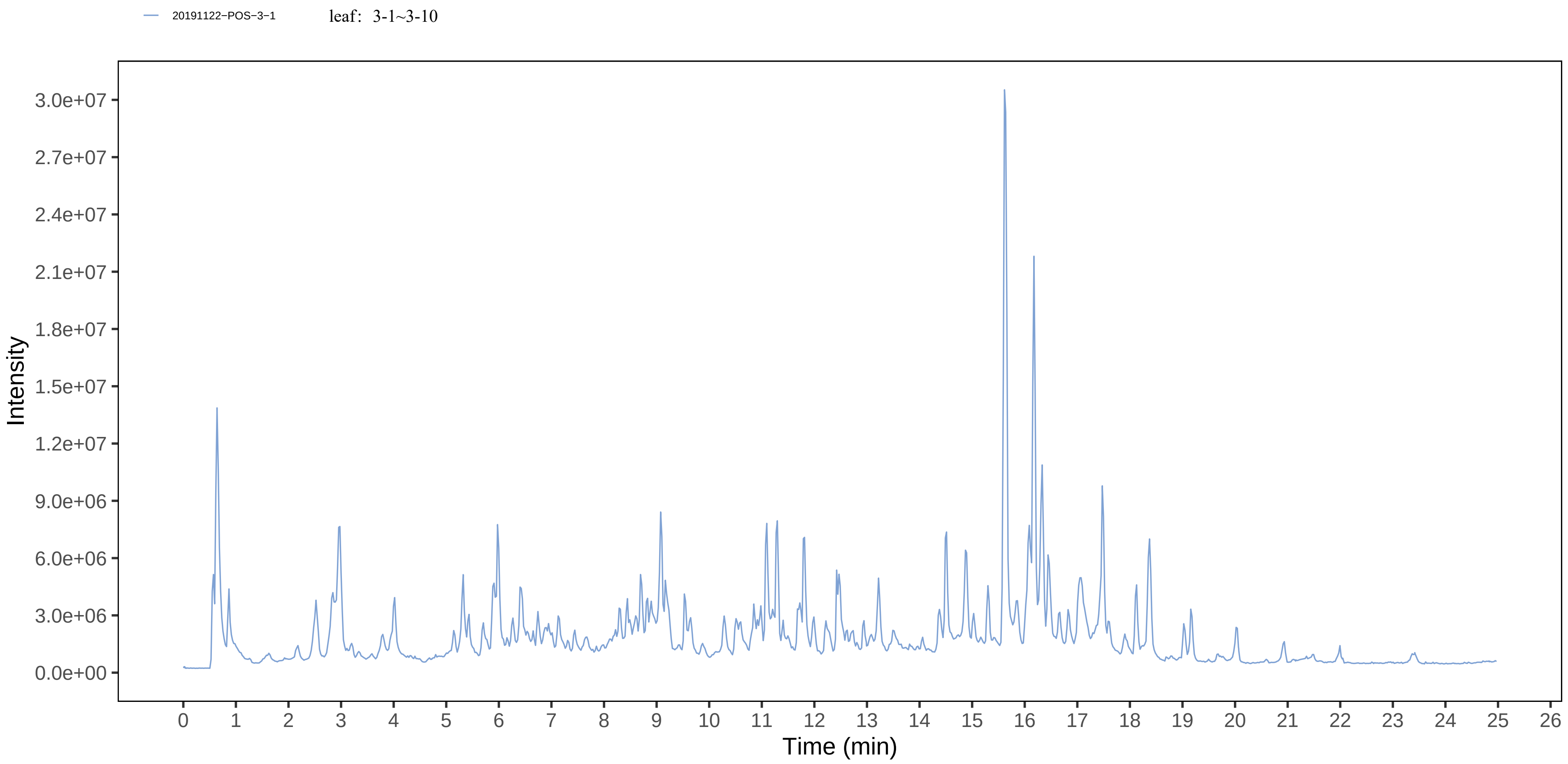
20191122−POS−3−1
leaf: 3-1~3-10
Intensity
3.0e+07
2.7e+07
2.4e+07
2.1e+07
1.8e+07
1.5e+07
1.2e+07
9.0e+06
6.0e+06
3.0e+06
0.0e+00
0 1 2 3 4 5 6 7 8 9 10 11 12 13 14 15 16 17 18 19 20 21 22 23 24 25 26
Time (min)

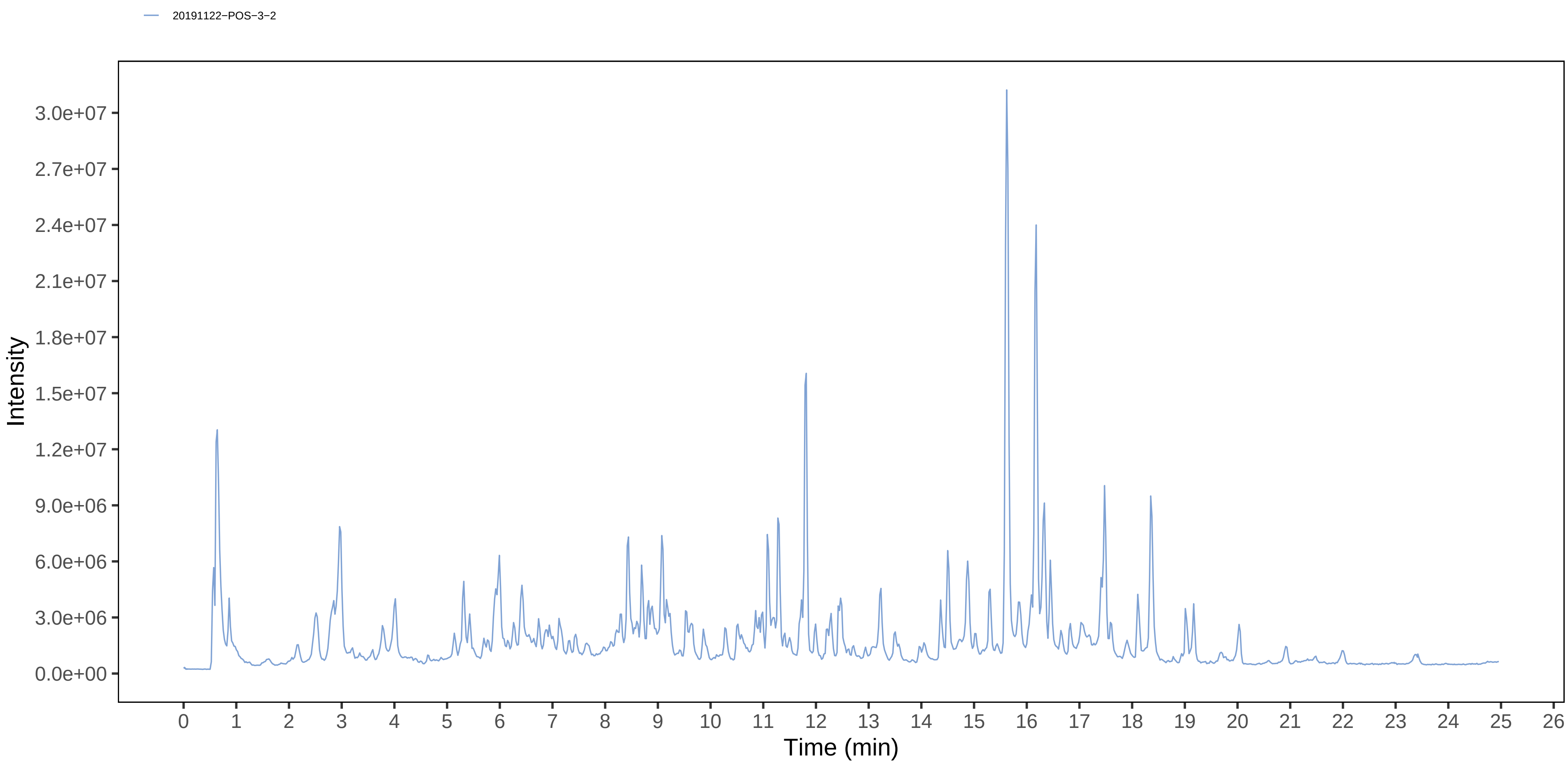

20191122−POS−3−2
Intensity
3.0e+07
2.7e+07
2.4e+07
2.1e+07
1.8e+07
1.5e+07
1.2e+07
9.0e+06
6.0e+06
3.0e+06
0.0e+00
0 1 2 3 4 5 6 7 8 9 10 11 12 13 14 15 16 17 18 19 20 21 22 23 24 25 26
Time (min)

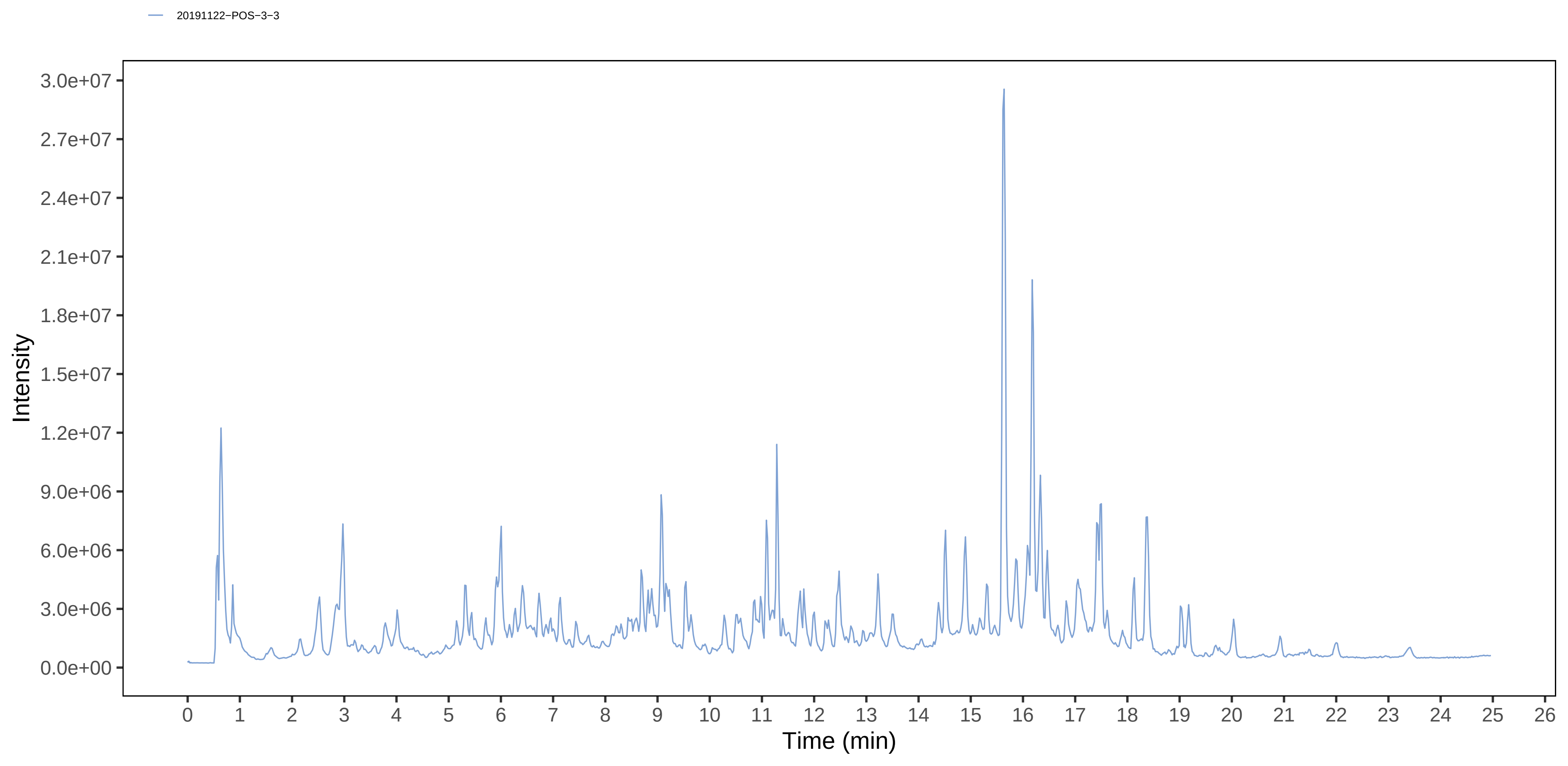

20191122−POS−3−3
Intensity
3.0e+07
2.7e+07
2.4e+07
2.1e+07
1.8e+07
1.5e+07
1.2e+07
9.0e+06
6.0e+06
3.0e+06
0.0e+00
0 1 2 3 4 5 6 7 8 9 10 11 12 13 14 15 16 17 18 19 20 21 22 23 24 25 26
Time (min)

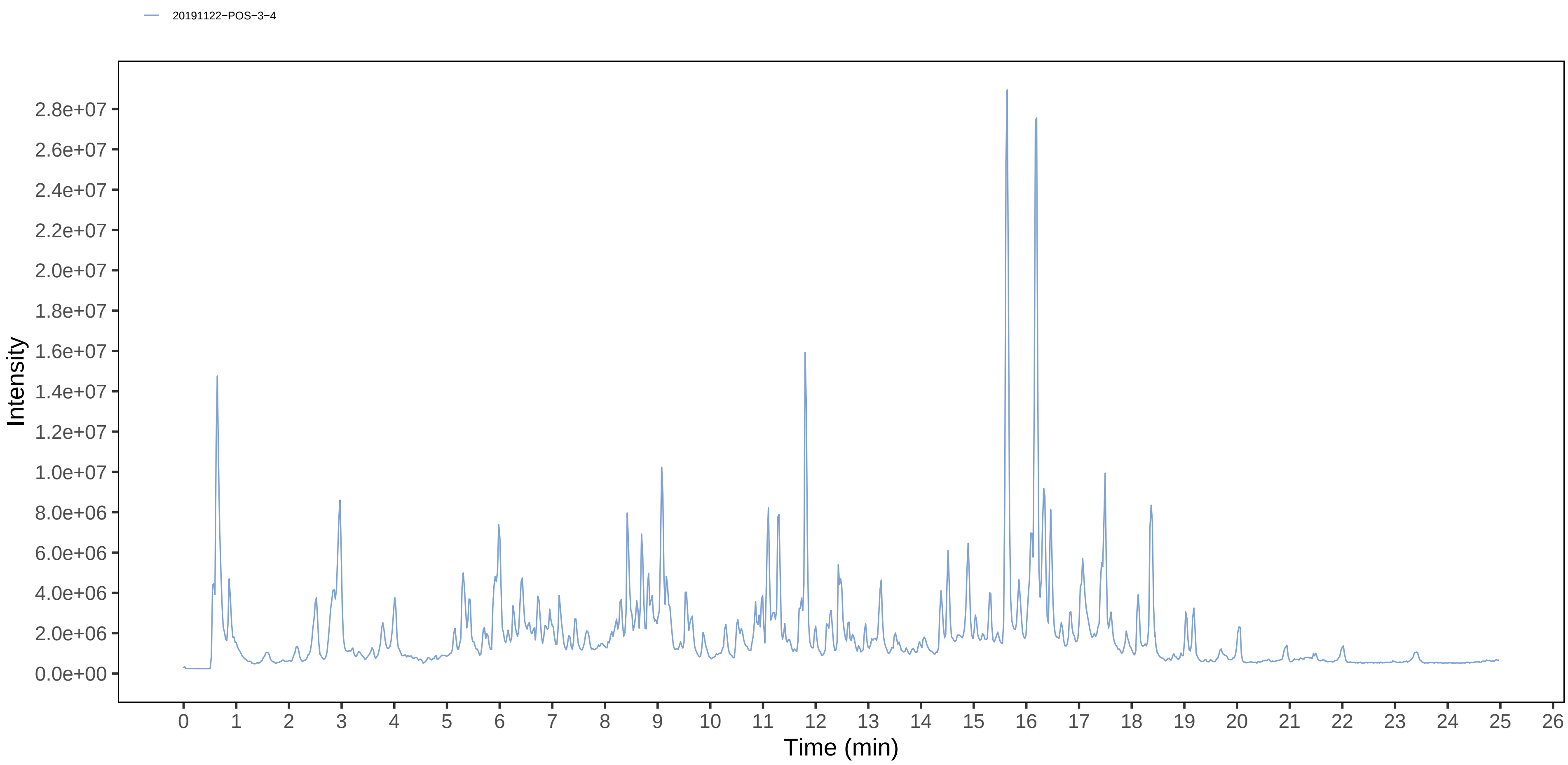
20191122−POS−3−4
Intensity
2.8e+07
2.6e+07
2.4e+07
2.2e+07
2.0e+07
1.8e+07
1.6e+07
1.4e+07
1.2e+07
1.0e+07
8.0e+06
6.0e+06
4.0e+06
2.0e+06
0.0e+00
0 1 2 3 4 5 6 7 8 9 10 11 12 13 14 15 16 17 18 19 20 21 22 23 24 25 26
Time (min)

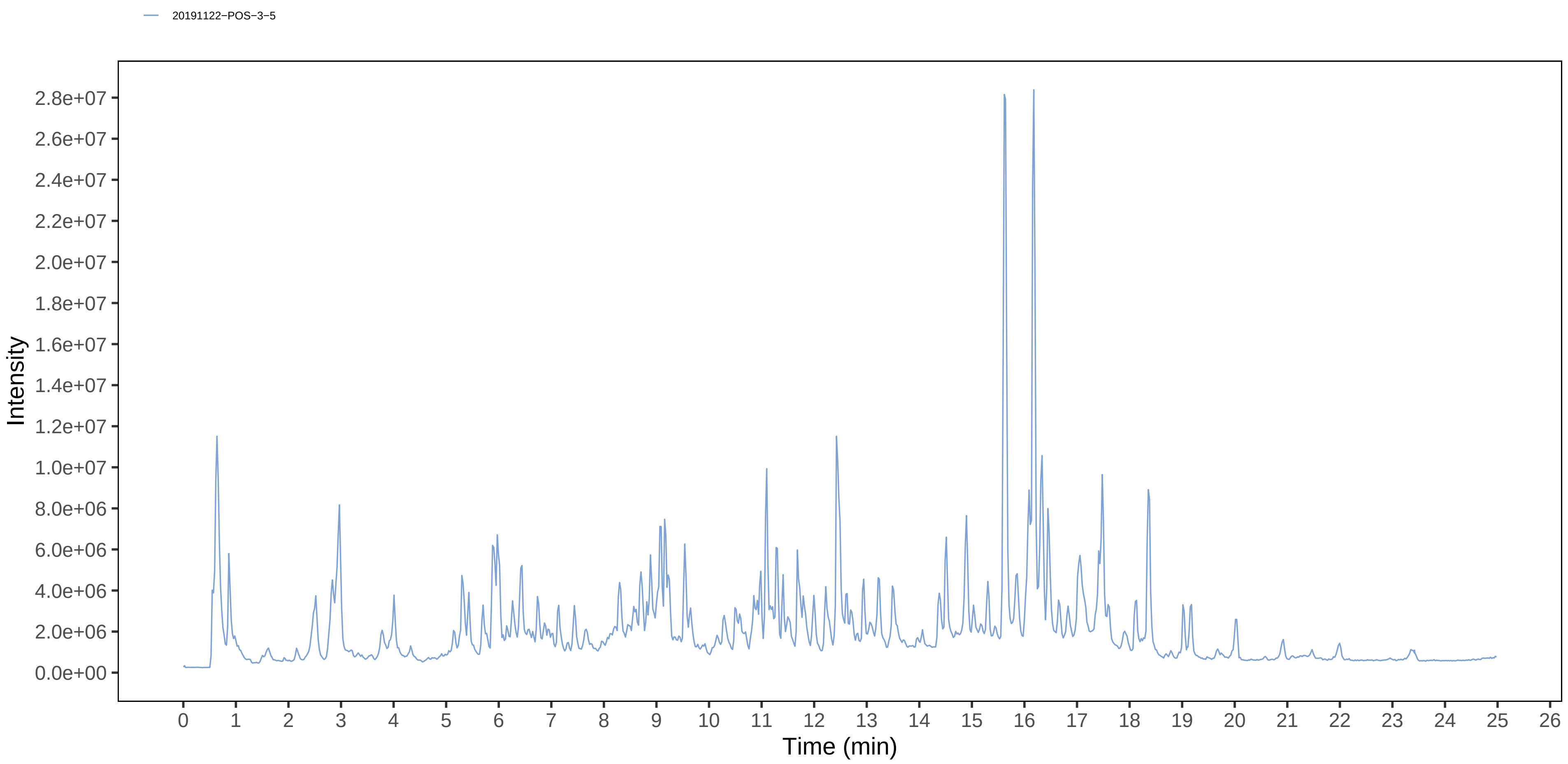
20191122−POS−3−5
Intensity
2.8e+07
2.6e+07
2.4e+07
2.2e+07
2.0e+07
1.8e+07
1.6e+07
1.4e+07
1.2e+07
1.0e+07
8.0e+06
6.0e+06
4.0e+06
2.0e+06
0.0e+00
0 1 2 3 4 5 6 7 8 9 10 11 12 13 14 15 16 17 18 19 20 21 22 23 24 25 26
Time (min)

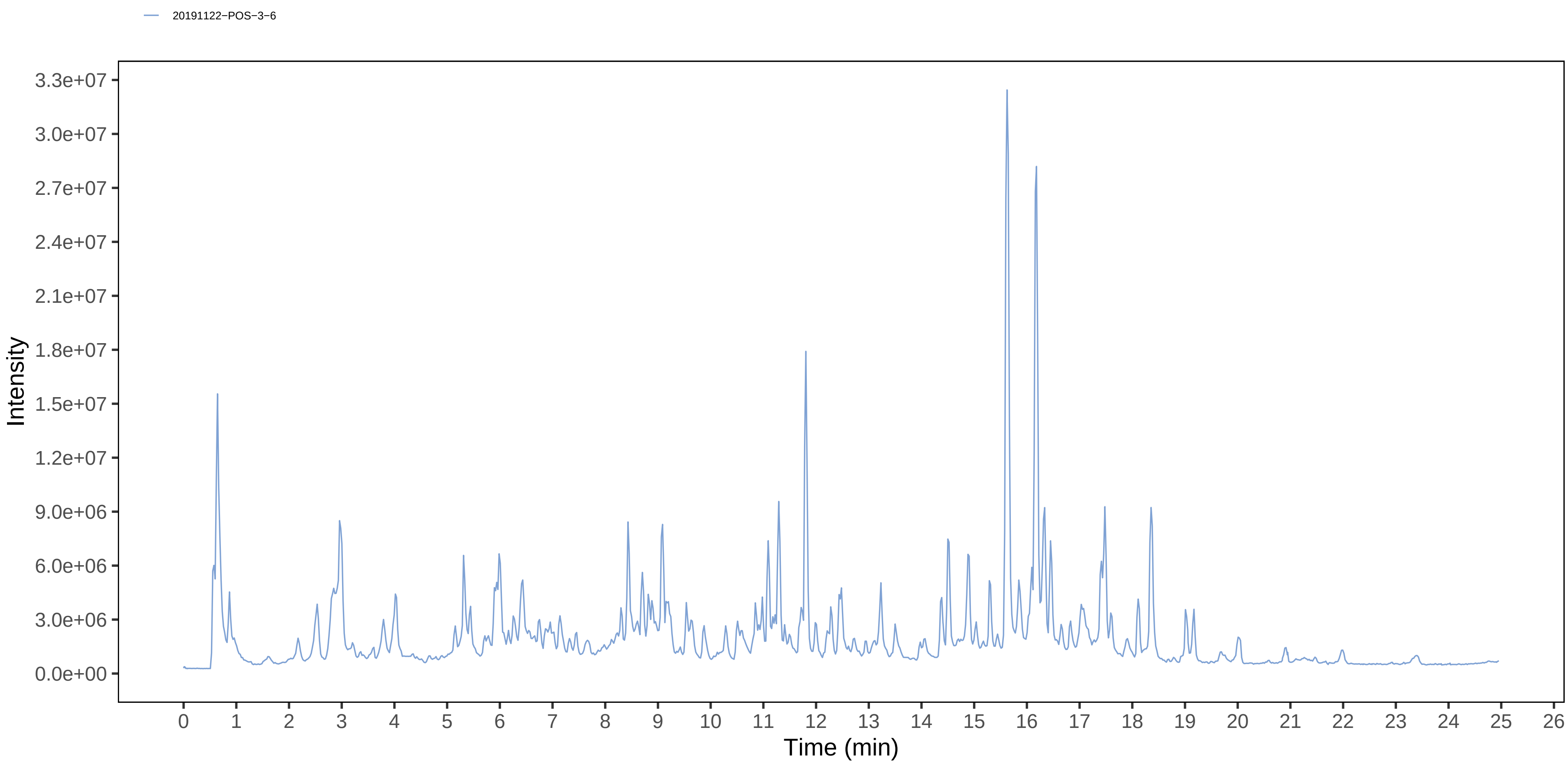

20191122−POS−3−6
Intensity
3.3e+07
3.0e+07
2.7e+07
2.4e+07
2.1e+07
1.8e+07
1.5e+07
1.2e+07
9.0e+06
6.0e+06
3.0e+06
0.0e+00
0 1 2 3 4 5 6 7 8 9 10 11 12 13 14 15 16 17 18 19 20 21 22 23 24 25 26
Time (min)

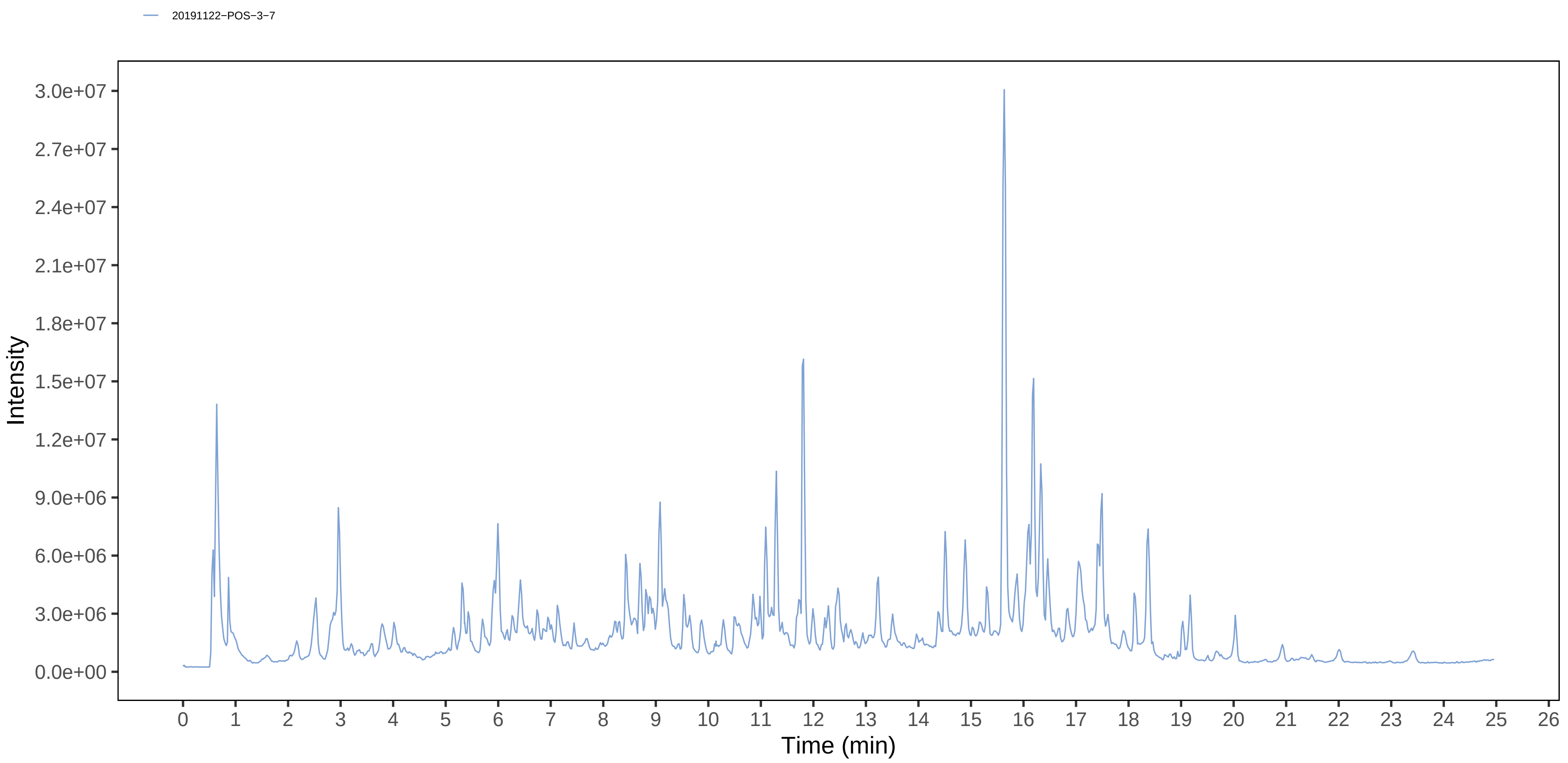

20191122−POS−3−7
Intensity
3.0e+07
2.7e+07
2.4e+07
2.1e+07
1.8e+07
1.5e+07
1.2e+07
9.0e+06
6.0e+06
3.0e+06
0.0e+00
0 1 2 3 4 5 6 7 8 9 10 11 12 13 14 15 16 17 18 19 20 21 22 23 24 25 26
Time (min)

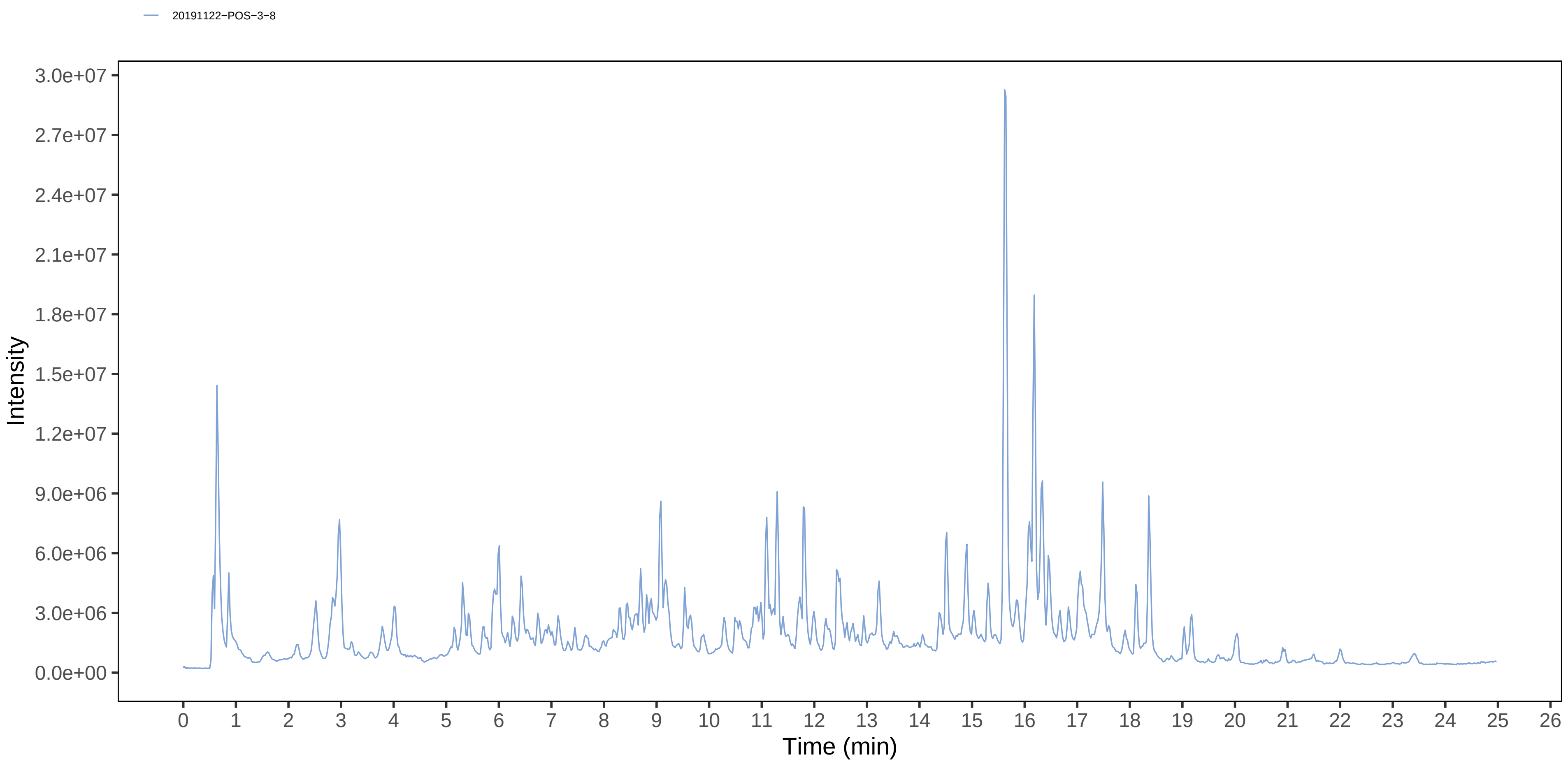

20191122−POS−3−8
Intensity
3.0e+07
2.7e+07
2.4e+07
2.1e+07
1.8e+07
1.5e+07
1.2e+07
9.0e+06
6.0e+06
3.0e+06
0.0e+00
0
1
2
3
4
5
6
7
8
9
10
11
12
13
14
15
16
17
18
19
20
21
22
23
24
25
26
Time (min)

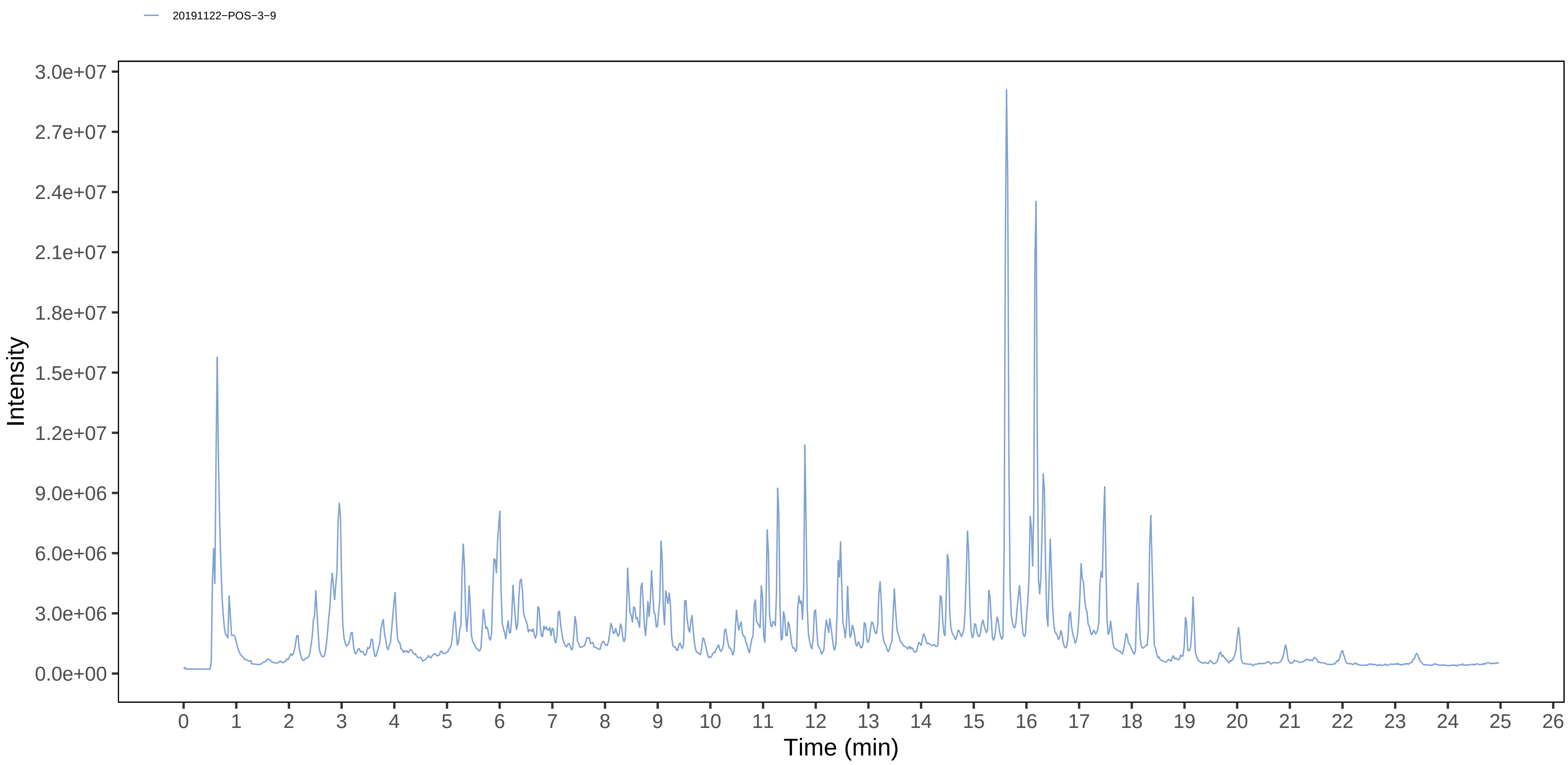

20191122−POS−3−9
Intensity
3.0e+07
2.7e+07
2.4e+07
2.1e+07
1.8e+07
1.5e+07
1.2e+07
9.0e+06
6.0e+06
3.0e+06
0.0e+00
0 1 2 3 4 5 6 7 8 9 10 11 12 13 14 15 16 17 18 19 20 21 22 23 24 25 26
Time (min)

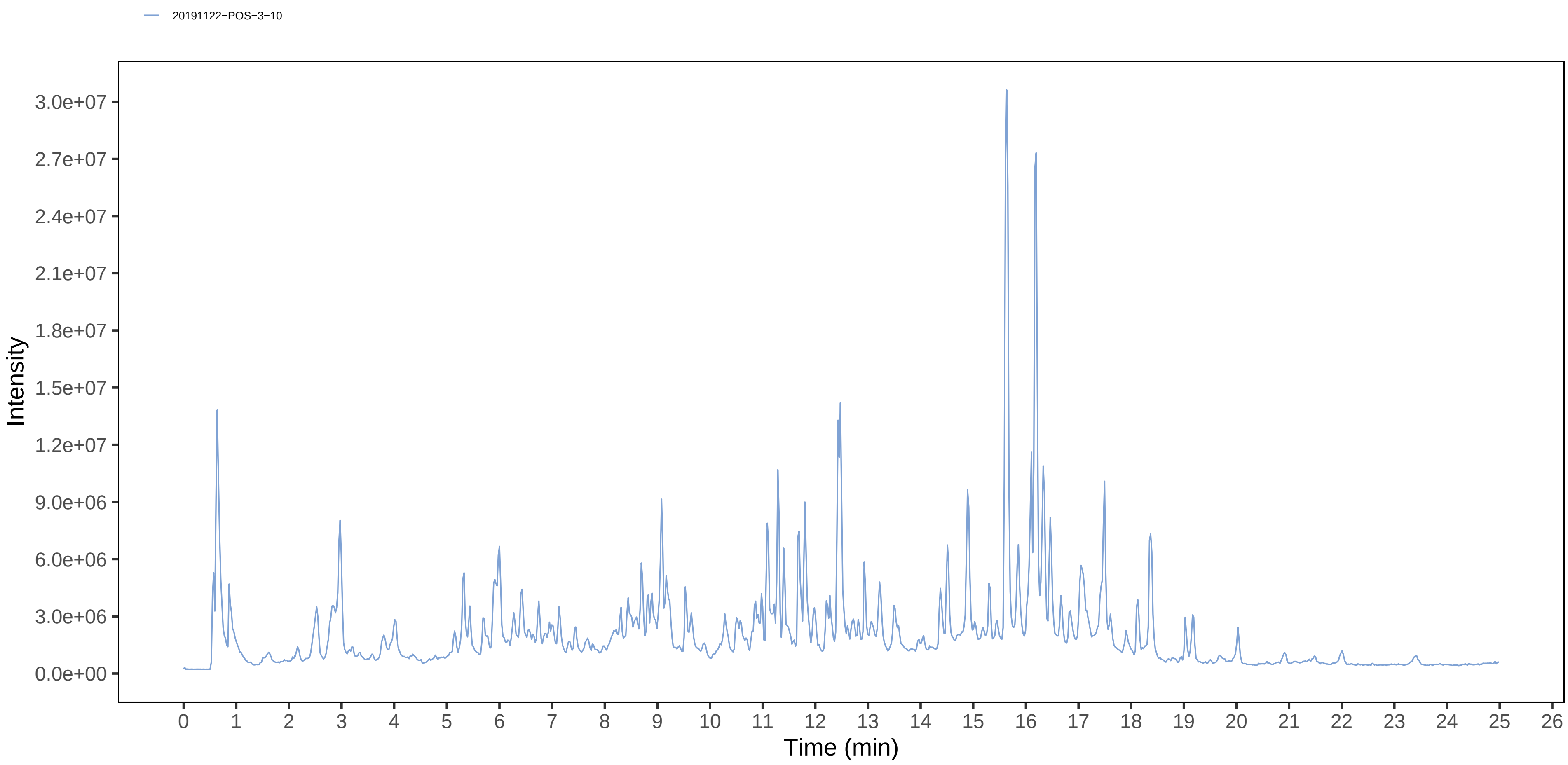
20191122−POS−3−10
Intensity
3.0e+07
2.7e+07
2.4e+07
2.1e+07
1.8e+07
1.5e+07
1.2e+07
9.0e+06
6.0e+06
3.0e+06
0.0e+00
0 1 2 3 4 5 6 7 8 9 10 11 12 13 14 15 16 17 18 19 20 21 22 23 24 25 26
Time (min)
