# Supplementary material for: Different Metabolites in the Roots, Seeds, and Leaves of Acanthopanax senticosus and Their Role in Alleviating Oxidative Stress
Source: J Anal Methods Chem. 2021 Apr 15;2021:6628880. doi: 10.1155/2021/6628880 (PMC8064801; doi:10.1155/2021/6628880)
Supplement: Supplementary Materials — Supplementary figure: TIC of all QC samples and TIC of different parts of A. senticosus. [file 6628880.f1.zip › 6628880.f1/Figures S3.docx]

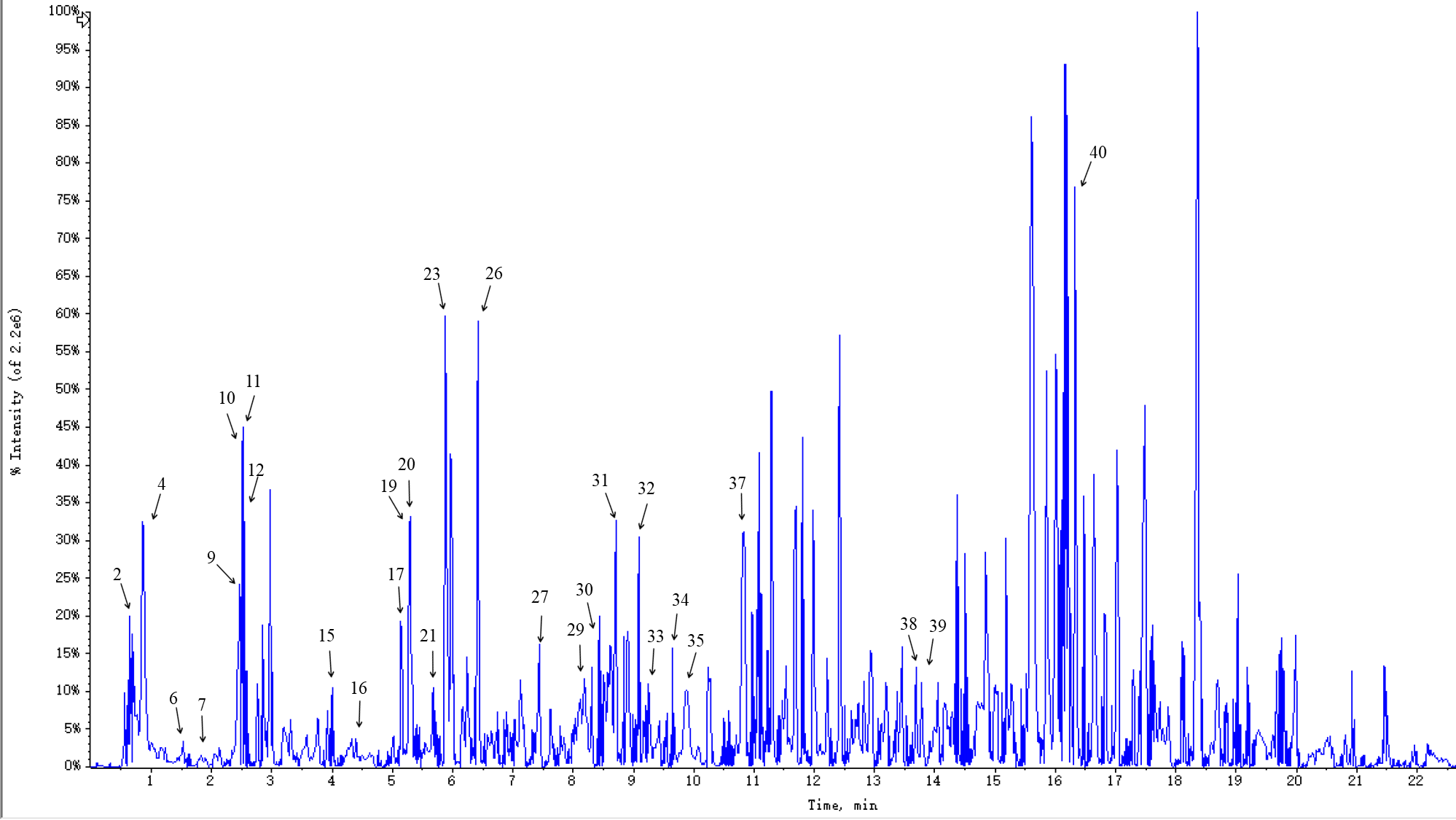

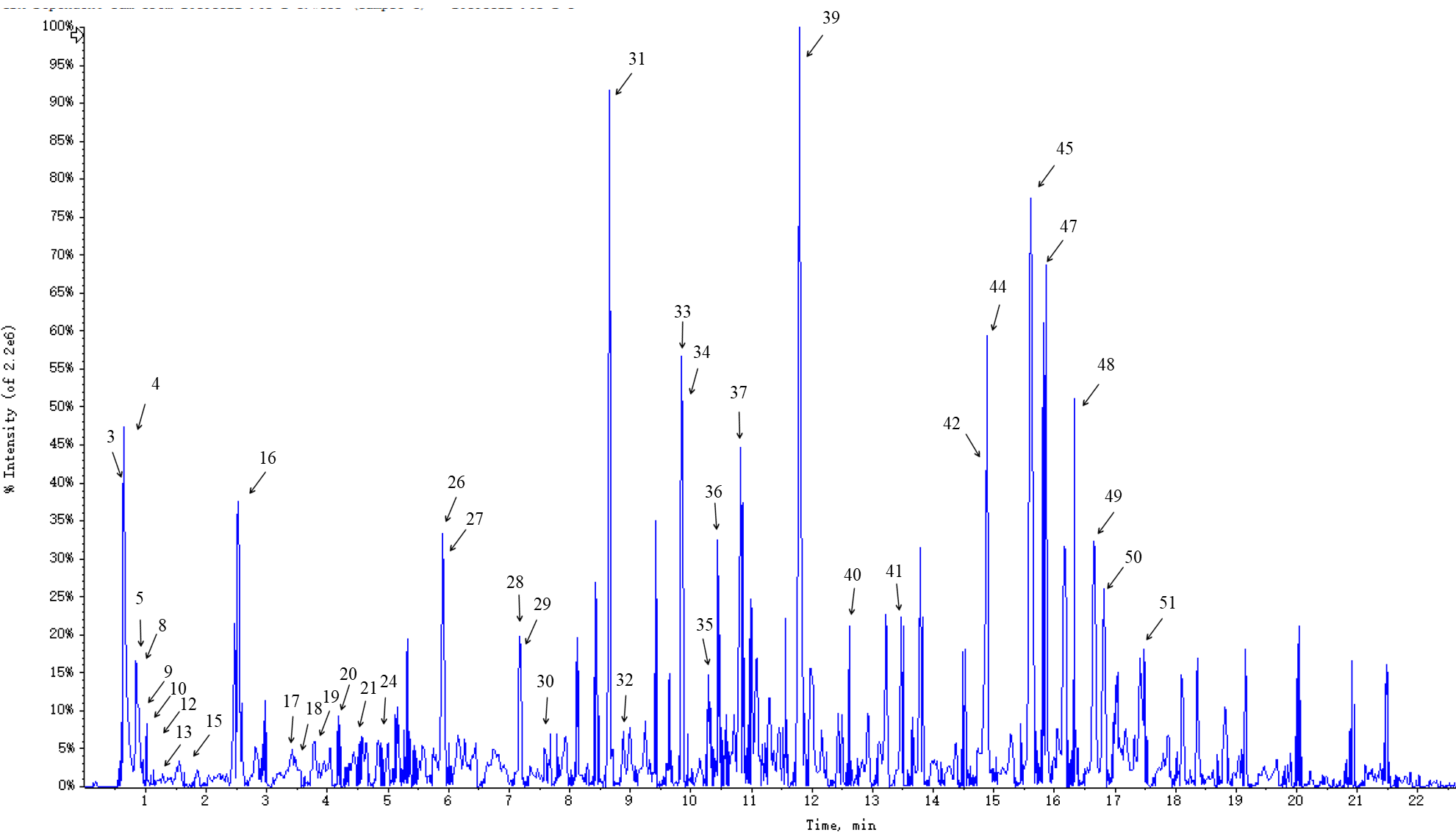

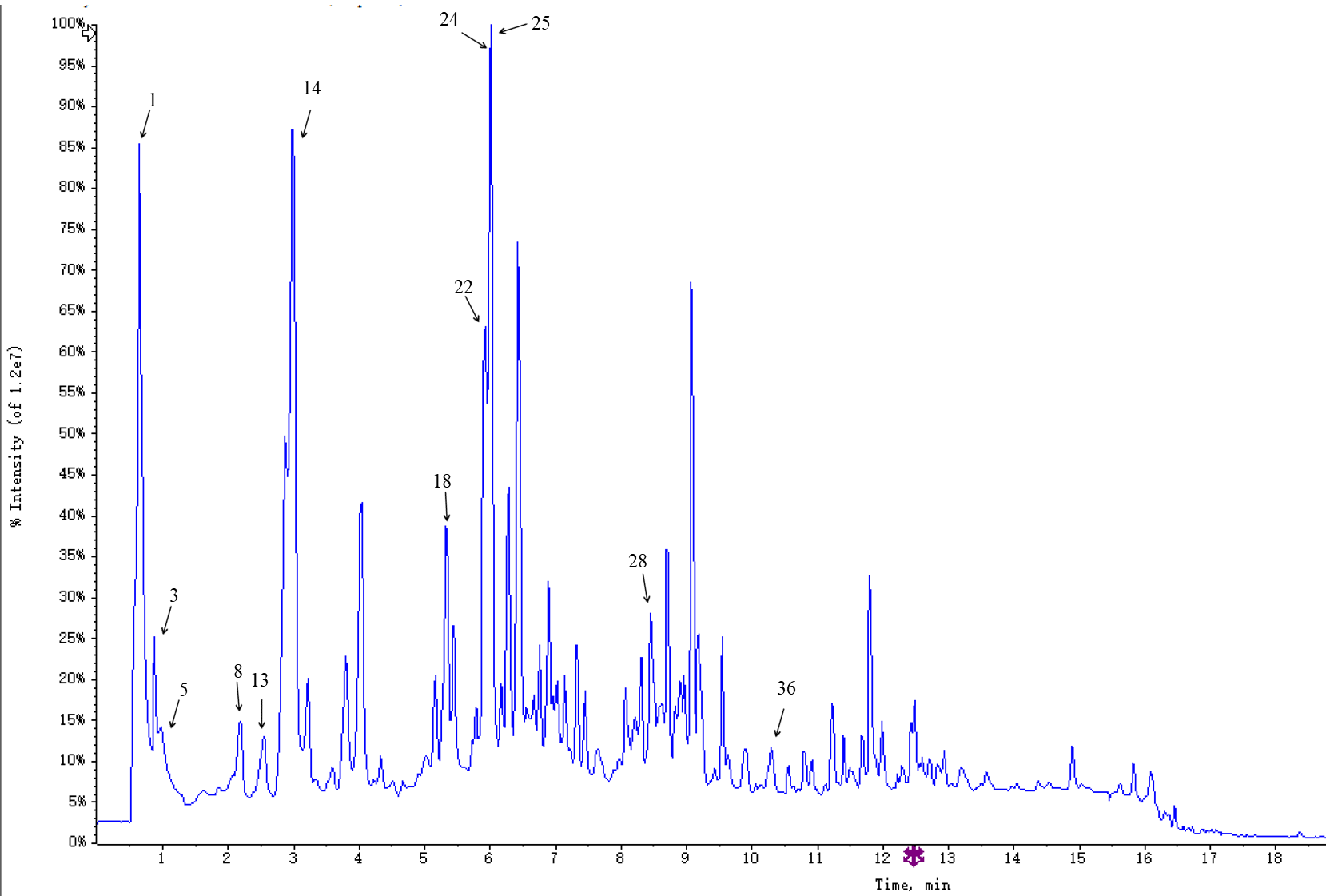

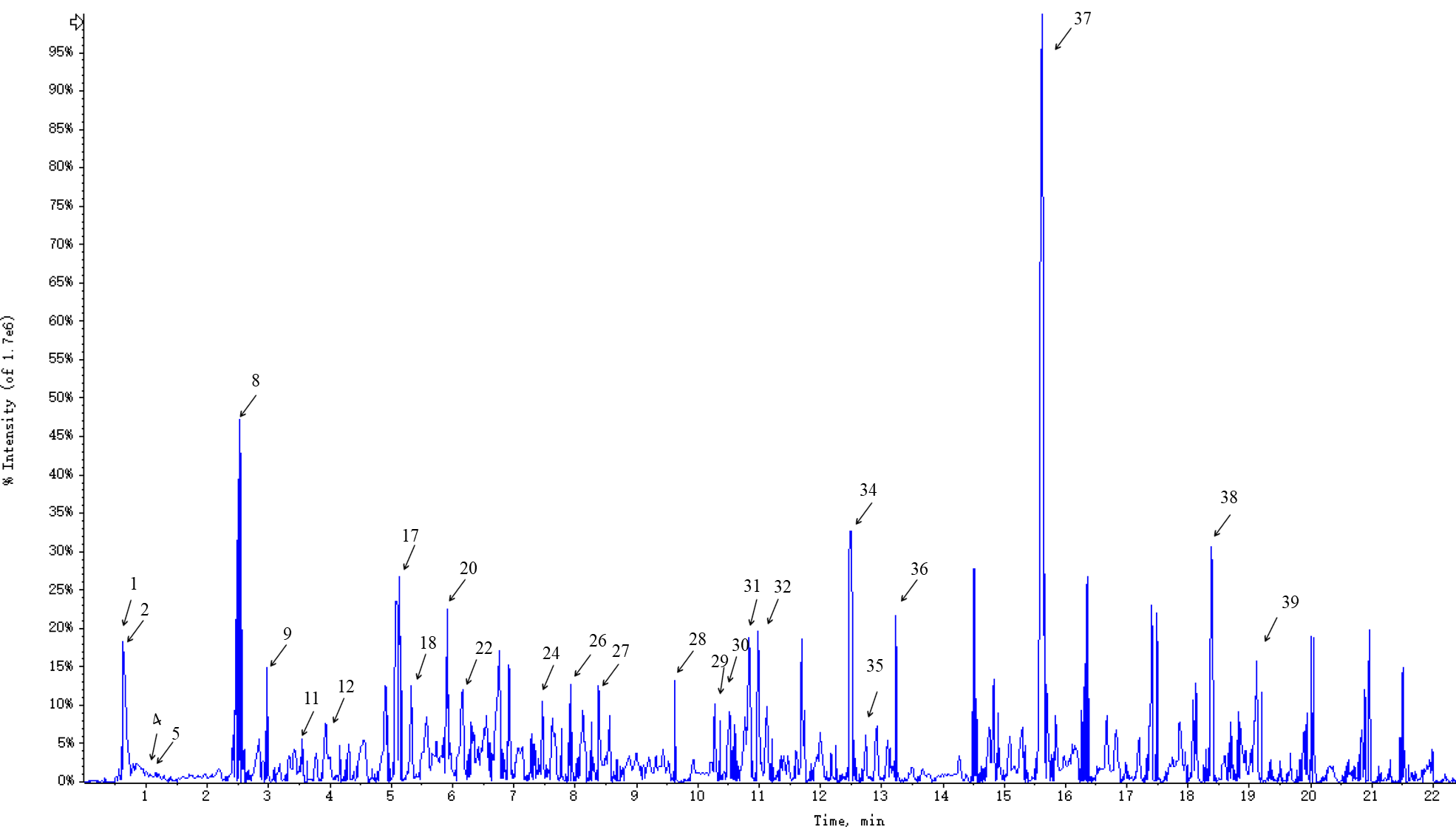

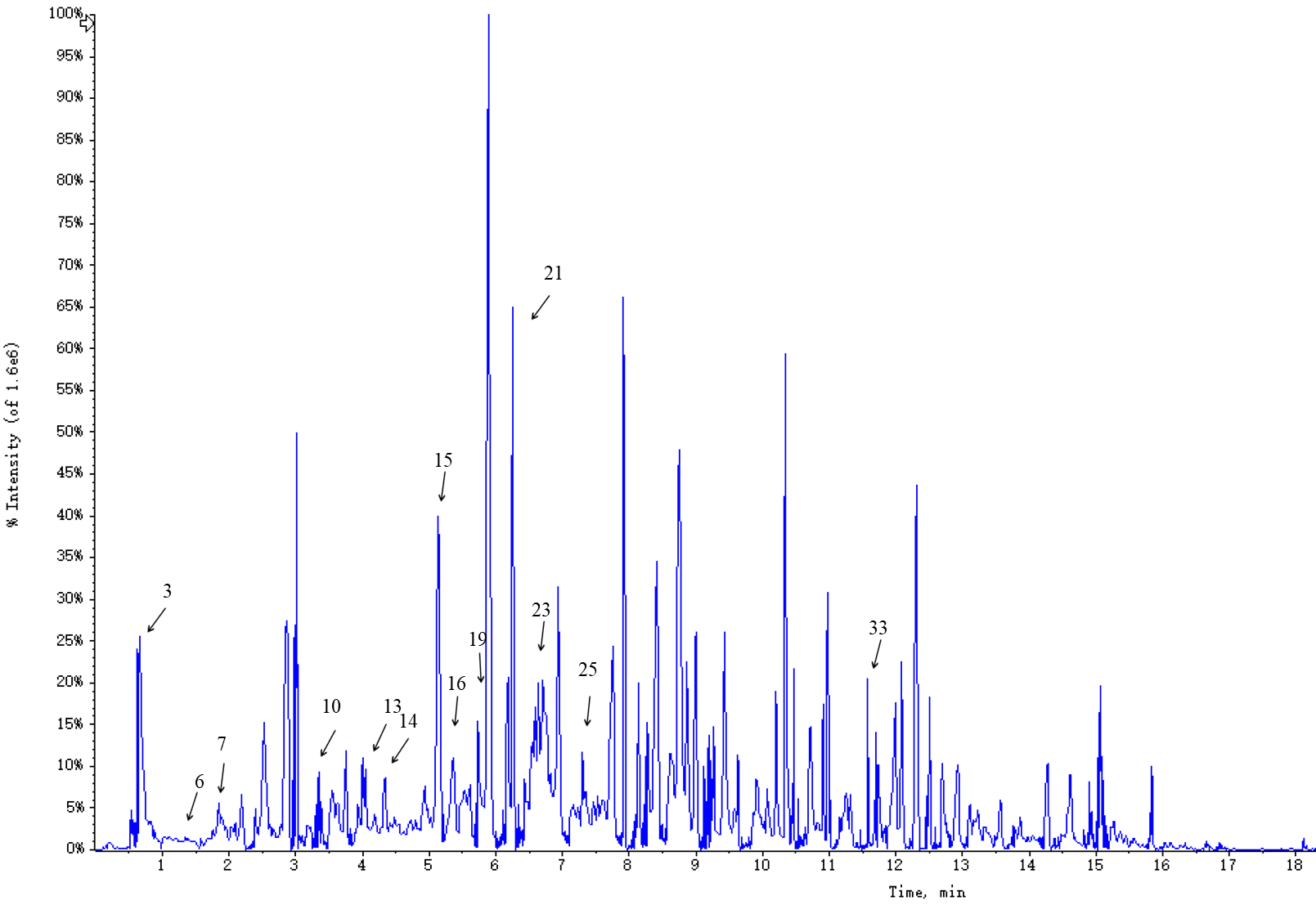

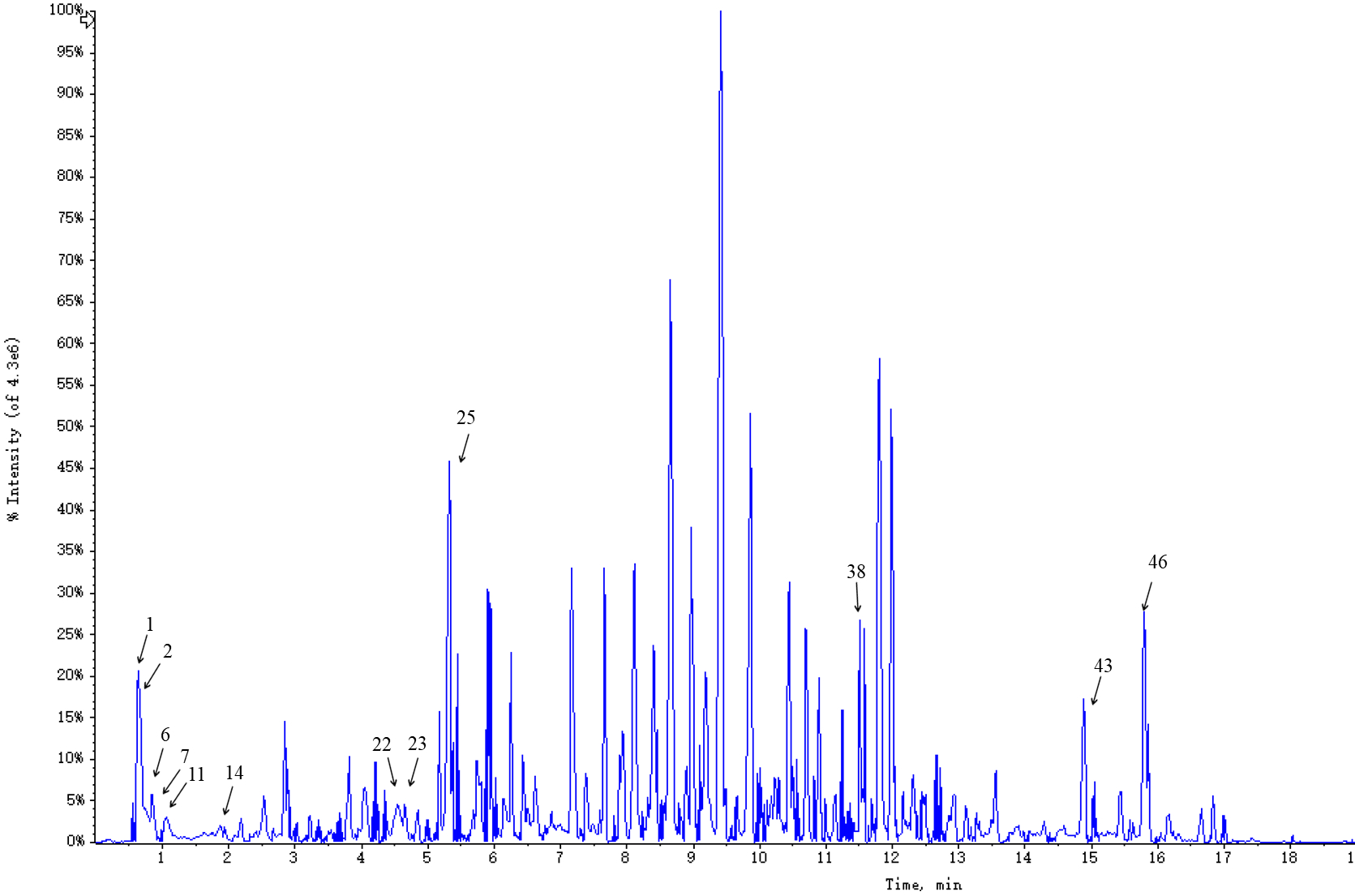


Note: Representative chromatograms of different metabolites from the different parts of A. senticosus in negative ion mode (-) and positive ion mode (+).

**Leaf (3-1)**

**Seed (2-1)**

**Root (1-1)**

**一**

**+**
