# Supplementary material for: Different Metabolites in the Roots, Seeds, and Leaves of Acanthopanax senticosus and Their Role in Alleviating Oxidative Stress
Source: J Anal Methods Chem. 2021 Apr 15;2021:6628880. doi: 10.1155/2021/6628880 (PMC8064801; doi:10.1155/2021/6628880)
Supplement: Supplementary Materials — Supplementary figure: TIC of all QC samples and TIC of different parts of A. senticosus. [file 6628880.f1.zip › 6628880.f1/Table S1.docx]

| **name** | **description** |
| --- | --- |
| id | The unique data number of the substance in this qualitative analysis |
| MS2 name | The name of the substance obtained by the qualitative matching analysis of the MS2 |
| MS1 name | The name of the first-level fragment ion matching, refer to the HMDB database or the KEGG database |
| m/z | mass-to-charge ratio |
| rt | Retention Time |

| id | MS2 name | MS1 name | mz | rt |
| --- | --- | --- | --- | --- |
| POS131 | (-)-Hibalactone | 10.84_352.0943n | 353.1015 | 10.84 |
| NEG60 | (-)-Quinic acid | 0.64_191.0557m/z | 191.0557 | 0.64 |
| POS130 | (-)-Riboflavin | 3.96_376.1377n | 377.145 | 3.96 |
| POS128 | (+)-Lirioresinol B | 6.17_418.1618n | 401.1585 | 6.17 |
| POS127 | .alpha.-Bisabolol | 13.49_222.1982n | 205.1949 | 13.49 |
| POS126 | .beta.-Sitosterol | 18.92_397.3820m/z | 397.382 | 18.92 |
| NEG58 | 1-(4-Hydroxy-3,5-dimethoxyphenyl)ethanone | 1.21_195.0656m/z | 195.0656 | 1.21 |
| POS119 | 1-Monolinoleoyl-rac-glycerol | 15.03_354.2764n | 377.2656 | 15.03 |
| POS123 | 10-Hydroxy-2-decenoic acid | 6.12_169.1223m/z | 169.1223 | 6.12 |
| POS122 | 12(13)-Epoxy-9Z-octadecenoic acid | 12.49_279.2317m/z | 279.2317 | 12.49 |
| POS121 | 13-Keto-9Z,11E-octadecadienoic acid | 12.74_294.2192n | 317.2085 | 12.74 |
| POS116 | 2'-Deoxyadenosine | 0.96_252.1091m/z | 252.1091 | 0.96 |
| POS115 | 2'-Hydroxy-4'-methoxyacetophenone | 8.59_167.0700m/z | 167.07 | 8.59 |
| NEG52 | 2-Furancarboxylic acid | 0.87_111.0086m/z | 111.0086 | 0.87 |
| POS114 | 2-Hydroxy-6-aminopurine | 0.94_152.0563m/z | 152.0563 | 0.94 |
| NEG51 | 2-Hydroxypalmitic acid | 15.04_271.2277m/z | 271.2277 | 15.04 |
| POS118 | 24-Hydroxychiisanogenin | 9.86_500.3124n | 523.3015 | 9.86 |
| POS109 | 3-Amino-2-naphthoic acid | 3.53_188.0706m/z | 188.0706 | 3.53 |
| POS108 | 3-Aminopentanoic acid | 0.64_118.0858m/z | 118.0858 | 0.64 |
| POS107 | 3-Coumaric acid | 4.67_147.0439m/z | 147.0439 | 4.67 |
| NEG49 | 3-Hydroxy-4-methoxycinnamic acid | 5.13_193.0507m/z | 193.0507 | 5.13 |
| POS106 | 3-Indoleacrylic acid | 2.43_187.0633n | 188.0706 | 2.43 |
| POS111 | 3,4,2',4',6'-Pentahydroxychalcone | 7.08_289.0707m/z | 289.0707 | 7.08 |
| POS110 | 3,5-Dicaffeoylquinic acid | 5.90_517.1329m/z | 517.1329 | 5.9 |
| POS105 | 4',5-Dihydroxy-7-methoxyflavanone | 9.93_287.0913m/z | 287.0913 | 9.93 |
| POS104 | 4-Ethynylaniline | 2.54_118.0648m/z | 118.0648 | 2.54 |
| POS103 | 4-Guanidinobutyric acid | 0.87_146.0921m/z | 146.0921 | 0.87 |
| POS102 | 4-Indolecarbaldehyde | 2.45_146.0599m/z | 146.0599 | 2.45 |
| POS101 | 6-Hydroxy-7-methoxycoumarin | 5.04_192.0422n | 193.0495 | 5.04 |
| POS100 | 6-Methylquinoline | 3.44_144.0805m/z | 144.0805 | 3.44 |
| POS99 | 7,8-Dihydro-.alpha.-ionone | 10.12_177.1638m/z | 177.1638 | 10.12 |
| POS97 | 9-Hydroperoxy-10E,12Z,15Z-octadecatrienoic acid | 10.44_293.2109m/z | 293.2109 | 10.44 |
| POS96 | 9-Oxo-10E,12Z-octadecadienoic acid | 12.94_294.2193n | 317.2086 | 12.94 |
| POS98 | 9E,11E-Octadecadienoic acid | 15.82_280.2400n | 263.2367 | 15.82 |
| NEG44 | Acanthoside B | 6.16_580.2151n | 579.2078 | 6.16 |
| POS89 | Anhuienside C | 8.87_1076.5986m/z | 1076.599 | 8.87 |
| NEG42 | Astragalin | 5.78_447.0925m/z | 447.0925 | 5.78 |
| POS87 | Avicularin | 5.65_434.0854n | 457.073 | 5.65 |
| NEG41 | Azelaic acid | 6.32_187.0976m/z | 187.0976 | 6.32 |
| NEG40 | Caffeic acid | 3.36_179.0352m/z | 179.0352 | 3.36 |
| POS84 | Caffeic acid methylester | 4.01_194.0578n | 217.0444 | 4.01 |
| NEG39 | Calenduloside E | 11.56_632.3918n | 631.3845 | 11.56 |
| NEG38 | Chiisanoside | 8.44_954.4818n | 999.4798 | 8.44 |
| NEG37 | Chlorogenic acid | 3.00_353.0874m/z | 353.0874 | 3 |
| POS82 | Chrysin | 9.86_255.0651m/z | 255.0651 | 9.86 |
| POS81 | cis-9-Hexadecenoic acid | 15.46_254.2246n | 237.2213 | 15.46 |
| POS80 | cis-Jasmone | 12.62_147.1168m/z | 147.1168 | 12.62 |
| POS79 | cis-Vaccenic acid | 16.84_282.2557n | 265.2524 | 16.84 |
| NEG36 | Citric acid | 0.87_191.0201m/z | 191.0201 | 0.87 |
| POS78 | Ciwujianoside C1 | 8.69_1042.5320n | 1065.521 | 8.69 |
| POS77 | Ciwujianoside D2 | 9.07_1084.5428n | 1107.532 | 9.07 |
| POS76 | Coniferin | 4.42_342.1313n | 365.12 | 4.42 |
| POS75 | Coniferyl aldehyde | 5.92_179.0702m/z | 179.0702 | 5.92 |
| POS74 | Curcumol | 10.57_219.1743m/z | 219.1743 | 10.57 |
| NEG33 | Cynarin | 5.92_515.1186m/z | 515.1186 | 5.92 |
| NEG32 | D-(+)-Galactose | 0.60_225.0619m/z | 225.0619 | 0.6 |
| NEG31 | D-(+)-Tryptophan | 2.46_203.0830m/z | 203.083 | 2.46 |
| NEG30 | D-Gluconic acid | 0.62_195.0511m/z | 195.0511 | 0.62 |
| POS72 | Dimethylfraxetin | 7.27_237.0757m/z | 237.0757 | 7.27 |
| POS71 | Dioctyl phthalate | 17.90_390.2764n | 413.2653 | 17.9 |
| NEG29 | DL-3-Phenyllactic acid | 4.88_165.0563m/z | 165.0563 | 4.88 |
| POS70 | DL-Arginine | 0.60_175.1187m/z | 175.1187 | 0.6 |
| POS69 | Echinocystic acid 3-glucoside | 10.25_657.3952m/z | 657.3952 | 10.25 |
| POS68 | Eleutheroside B | 3.15_395.1307m/z | 395.1307 | 3.15 |
| POS67 | Eleutheroside E | 5.13_742.2653n | 765.2558 | 5.13 |
| POS66 | Epicatechin | 2.70_291.0860m/z | 291.086 | 2.7 |
| NEG26 | Eriodictyol-7-O-glucoside | 5.48_449.1079m/z | 449.1079 | 5.48 |
| POS62 | Esculin | 2.45_340.0800n | 341.0873 | 2.45 |
| POS61 | Ethyl 2,4-dihydroxy-6-methylbenzoate | 3.49_196.0732n | 219.0628 | 3.49 |
| POS60 | Farnesal | 10.16_203.1793m/z | 203.1793 | 10.16 |
| NEG25 | Fraxidin | 4.52_221.0457m/z | 221.0457 | 4.52 |
| NEG24 | Fraxinol | 5.37_221.0458m/z | 221.0458 | 5.37 |
| NEG23 | Fumaric acid | 0.98_115.0036m/z | 115.0036 | 0.98 |
| NEG22 | Genkwanin | 10.03_283.0606m/z | 283.0606 | 10.03 |
| NEG21 | Gentisic acid | 1.92_153.0195m/z | 153.0195 | 1.92 |
| NEG20 | Guanosine | 0.94_282.0844m/z | 282.0844 | 0.94 |
| POS58 | Hederagenin | 9.21_472.3542n | 455.3509 | 9.21 |
| POS57 | Hemsgiganoside B | 8.62_956.4960n | 979.4854 | 8.62 |
| POS56 | Heptadecasphing-4-enine | 11.18_286.2733m/z | 286.2733 | 11.18 |
| POS55 | Heptadecasphinganine | 11.20_288.2897m/z | 288.2897 | 11.2 |
| NEG19 | Hexadecanedioic acid | 11.74_285.2071m/z | 285.2071 | 11.74 |
| NEG18 | Hyperoside | 5.33_463.0875m/z | 463.0875 | 5.33 |
| POS54 | Isofraxidin | 5.35_222.0537n | 223.061 | 5.35 |
| POS52 | L-2-Aminoadipic acid | 1.17_144.0652m/z | 144.0652 | 1.17 |
| POS48 | L-Leucine | 1.05_132.1016m/z | 132.1016 | 1.05 |
| POS47 | L-Phenylalanine | 1.63_165.0788n | 166.0861 | 1.63 |
| POS45 | L-Pyroglutamic acid | 0.87_129.0424n | 130.0496 | 0.87 |
| POS44 | L-Tyrosine | 0.89_182.0809m/z | 182.0809 | 0.89 |
| POS51 | Linarin | 7.29_592.1782n | 593.1855 | 7.29 |
| NEG17 | Linoleic acid | 15.81_280.2400n | 279.2327 | 15.81 |
| POS49 | Linoleic acid methyl ester | 17.61_294.2554n | 295.2627 | 17.61 |
| POS42 | Matairesinol | 8.25_358.1410n | 381.1303 | 8.25 |
| POS41 | Methyl .gamma.-linolenate | 16.82_292.2400n | 293.2473 | 16.82 |
| POS40 | Methyl 4-hydroxycinnamate | 1.10_179.0702m/z | 179.0702 | 1.1 |
| POS39 | Monolinolenin (9c,12c,15c) | 14.06_352.2601n | 375.2501 | 14.06 |
| POS38 | Morin | 5.33_303.0499m/z | 303.0499 | 5.33 |
| NEG15 | N-Acetyl-DL-glutamic acid | 0.89_188.0566m/z | 188.0566 | 0.89 |
| POS36 | N-Acetyl-L-glutamic acid | 0.89_189.0640n | 212.053 | 0.89 |
| NEG14 | N-Acetyl-L-phenylalanine | 4.79_206.0824m/z | 206.0824 | 4.79 |
| POS33 | N-Desmethyl-loperamide | 7.08_462.2066n | 463.183 | 7.08 |
| POS29 | N-Oleoylethanolamine | 15.10_325.2983n | 348.2879 | 15.1 |
| POS34 | Naringenin-7-O-glucoside | 6.15_434.1204n | 435.1271 | 6.15 |
| NEG13 | Neochlorogenic acid | 2.19_353.0873m/z | 353.0873 | 2.19 |
| POS32 | Niacinamide | 0.87_123.0551m/z | 123.0551 | 0.87 |
| POS31 | Nipponoside B | 8.55_1088.5771n | 1111.563 | 8.55 |
| NEG11 | Nitrobenzene | 1.78_122.0246m/z | 122.0246 | 1.78 |
| POS28 | Nootkatone | 12.01_201.1637m/z | 201.1637 | 12.01 |
| POS27 | Norharmane | 4.14_169.0760m/z | 169.076 | 4.14 |
| POS26 | Oleic acid | 16.32_282.2558n | 265.2525 | 16.32 |
| NEG9 | p-Coumaric acid | 4.42_163.0403m/z | 163.0403 | 4.42 |
| POS20 | p-Hydroxyphenyllactic acid | 0.91_165.0543m/z | 165.0543 | 0.91 |
| POS24 | Palmitamide | 15.31_255.2562n | 256.2634 | 15.31 |
| POS23 | Pantothenic acid | 1.81_219.1107n | 220.1179 | 1.81 |
| POS22 | Pheophorbide a | 16.18_592.2665n | 593.2738 | 16.18 |
| POS18 | Pinolenic acid | 14.06_261.2212m/z | 261.2212 | 14.06 |
| POS17 | Pinoresinol 4-O-glucoside | 6.58_520.1931n | 543.1824 | 6.58 |
| POS14 | Quercetin | 7.20_303.0497m/z | 303.0497 | 7.2 |
| NEG8 | Quercitrin | 6.01_447.0923m/z | 447.0923 | 6.01 |
| POS12 | Rutin | 5.26_611.1595m/z | 611.1595 | 5.26 |
| NEG6 | Sebacic acid | 7.31_202.1209n | 201.1134 | 7.31 |
| POS11 | Serotonin | 2.45_159.0916m/z | 159.0916 | 2.45 |
| POS10 | Sesamin | 10.98_354.1098n | 355.1171 | 10.98 |
| POS9 | Spiraeoside | 5.33_465.1019m/z | 465.1019 | 5.33 |
| NEG4 | Suberic acid | 5.03_173.0822m/z | 173.0822 | 5.03 |
| NEG3 | Succinic acid | 0.96_117.0193m/z | 117.0193 | 0.96 |
| POS8 | Sucrose | 0.69_342.1158n | 365.105 | 0.69 |
| POS5 | Valerenic acid | 10.39_234.1618n | 235.1691 | 10.39 |
| POS4 | Vanillin | 4.30_153.0543m/z | 153.0543 | 4.3 |
| POS3 | Verrucarol | 9.23_266.1516n | 289.1409 | 9.23 |
| POS1 | Xanthurenic acid | 1.85_206.0451m/z | 206.0451 | 1.85 |
